# Characterization of Phyllobilins in Hops: Antioxidant and Potentially Bitter Senescence-Related Metabolites

**DOI:** 10.1021/acs.jafc.5c03549

**Published:** 2025-07-07

**Authors:** Christian Nadegger, Patricia Frei, Christian A. Elvert, Cornelia A. Karg, Johanna M. Gostner, Jonathan S. Lindsey, Christoph R. Kreutz, Stefan Schwaiger, Thomas Müller, Simone Moser

**Affiliations:** † Institute of Organic Chemistry, University of Innsbruck, Innrain 80/82, A-6020 Innsbruck, Austria; ‡ Center for Molecular Biosciences, University of Innsbruck, Innrain 80/82, A-6020 Innsbruck, Austria; § Department of Pharmacy, University of Munich, Butenandtstraße 5-13, D-81377 Munich, Germany; ∥ Department of Pharmacognosy, Institute of Pharmacy, University of Innsbruck, Innrain 80/82, A-6020 Innsbruck, Austria; ⊥ Institute of Medical Biochemistry, Medical University of Innsbruck, Innrain 80/82, A-6020 Innsbruck, Austria; # Department of Chemistry, North Carolina State University, Raleigh, North Carolina 27695, United States

**Keywords:** chlorophyll, plant senescence, antioxidant, natural products, mass spectrometry

## Abstract

Hops is of high relevance to the food sector, and increasingly valued as medicinal plant. Its complex phytochemistry includes phenolic compounds and bitter prenylated polyketides, but phyllobilins—bioactive linear tetrapyrroles from chlorophyll catabolism—remain underexplored. In this work, several dioxobilin-type phylloleucobilins (DPleBs) and phylloxanthobilins (DPxBs) were identified in yellowish leaves of common hops (*Humulus lupulus*). Isolation from 107 g of leaves yielded 0.24 mg of *Hl*-DPleB-28 and 0.80 mg of *Hl*-DPxB-31. Structural elucidation via UV/vis, HR-MS^2^, and NMR confirmed those as new phyllobilins, featuring an unusual hydroxylation motif, indicating an uncharacterized metabolic pathway. *Hl*-DPxB constituted about 40% of HPLC peak areas at 420 nm in yellow leaves, suggesting its significant role in the visual senescence of hops. *Hl*-DPxB-31 possessed high antioxidative activity, comparable to quercetin. A virtual tool predicted over 60% bitterness probability. These findings expand the phytochemical profile of hops and highlight potential for upcycling leaf waste.

## Graphical abstract

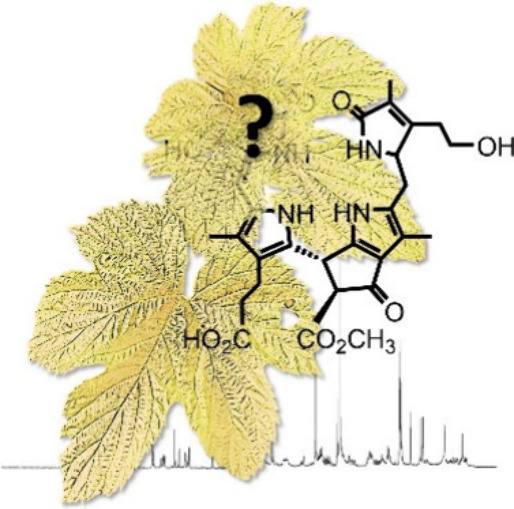

## Introduction

Hops (*Humulus lupus* L., Cannabaceae) are widely known as constituents for brewing beer, with pollen evidence pointing to use since the Roman era. The female cones of the hop plant are used, which impart a bitter sensation as well as a preservative effect to beer. The cones are also used medicinally to treat mild sleeping disorders and temporary insomnia or mental stress and mood disorders.^1^ Both effects can be traced to the presence of unique secondary metabolites known as bitter acids (prenylated phloroglucinol derivatives) and a mixture of prenylated, geranylated, oxidized, and/or cyclized chalcones, as well as an essential oil.^2^ Bitter compounds in hops are also relevant for the taste of hop products and hence for beer brewing, and have been the focus of recent research.^3,4^ Although the plant is perennial, only the underground parts can withstand the cold season whereas the aerial parts are renewed each year as fast-growing climbing vines (up to 10 m). Similar to asparagus, the young shoots that emerge in early spring are consumed as vegetables in several European countries. Given that the global hop harvest reached in 2019 already 131 kilotons and only a small part of the aerial biomass is actually used, the remaining material offers potential for additional-value byproducts^5^ including primary metabolites or use as fibers. Therefore, the search for bioactive constituents of hops as a highly relevant agricultural plant is of utmost importance for opening incentives and possibilities for more sustainable use. In this study, the aim was to investigate one often neglected metabolite group in plant tissues, bilin-type degradation products of chlorophyll, the phyllobilins (PBs), which are known to be remarkable and ubiquitous antioxidants that can be found especially in cells of aging leaves and ripening fruits.^6^ The chemical composition typically changes during senescence, and for agriculturally relevant plants, such as hops, the possibility of a late harvest to increase certain flavors and aromas, based on volatile thiols, is an attractive approach that needs thorough phytochemical profiling.^7^

PBs have been found to occur with high structural diversity across the plant kingdom,^8^ resulting from presumably enzymatic modifications of the tetrapyrrole core. To date, only a limited number of positions have been discovered that are chemically modified in the course of chlorophyll degradation (Figure 1). Considering the fact that chlorophyll degradation is associated with intense coloration of leaves in autumn, it was surprising at first that the earlier identified metabolites turned out to be linear, colorless tetrapyrroles.^9^ Nowadays, these colorless PBs are termed phylloleucobilins (PleBs).^10^ In the meantime, yellow colored phyllobilins have been identified and characterized, and have been shown to contribute to the autumn colors.^11^ The yellow pigments are now called phylloxanthobilins (PxBs). Later, a second lineage of PBs was discovered and termed type-II PBs, or dioxobilin-type PBs, which result from the activity of a cytochrome enzyme.^12^ Also for the type-II PB lineage, colorless (DPleBs) and yellow (DPxBs) metabolites have been reported.^8^

**Figure 1:**
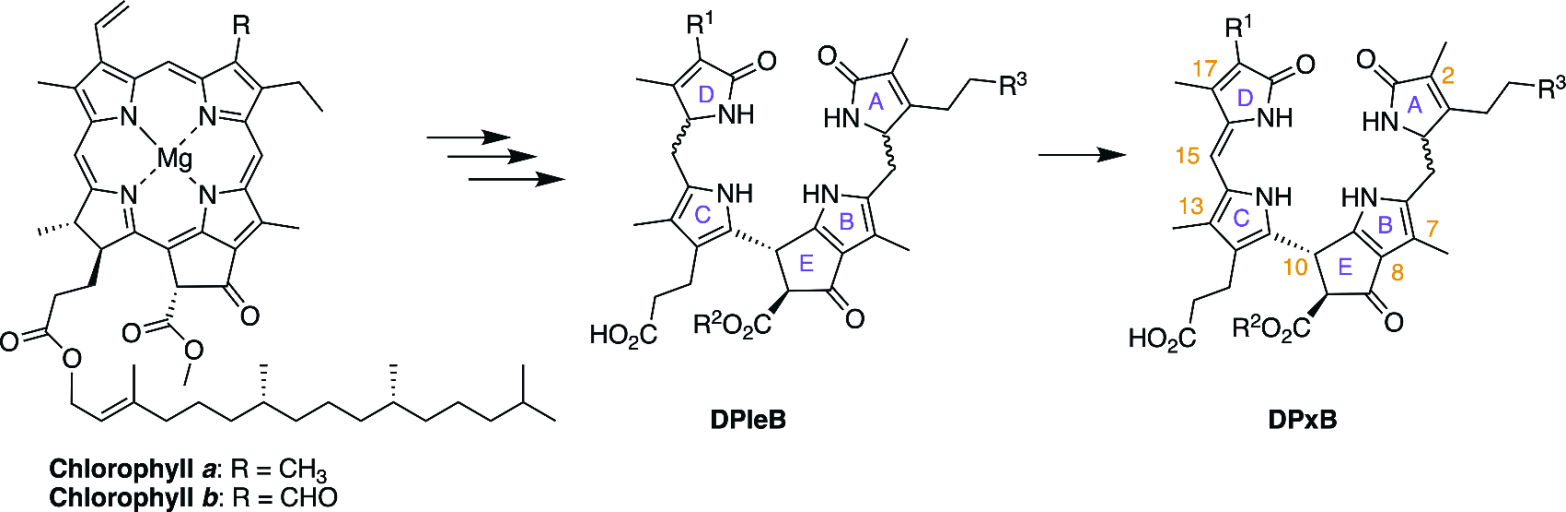
Structures of Chls a and b (left), and of dioxobilin-type phyllobilins, a phylloleucobilin (DPleB, middle), and a phylloxanthobilin (DPxB, right). Those modification sites known to date in a dioxobilin-type chromophore are indicated by R_1–3_. Atom- and ring-numbering in PBs are shown in the DPxB structure.

In a further shift away from the perception of PBs as mere throw-away products from the degradation of chlorophyll, recent research has uncovered potent bioactivities for PBs that include antioxidative,^13^ anti-inflammatory,^14,15^ and anticancer activities.^16,17^ Hence, PBs are emerging as bioactive phytochemicals that might play a role in the efficacies of phytotherapies as well as health benefits of plant-based nutrition.^6,18^ In this respect, type-I PBs have been studied more thoroughly to date compared to the type-II lineage. Plants with nutritional or phytotherapeutic use containing type-II PBs are therefore interesting not only as new sources of this understudied type of natural products, but also as an opportunity to access these compounds, and to identify new structures and bioactivities. Hence, the present study aimed at identifying, isolating, and structurally characterizing phyllobilins from senescent hop (*Humulus lupulus*) leaves, and to evaluate their contribution to leaf coloration, antioxidant activity, and potential bitterness, thereby expanding the understanding of chlorophyll catabolite diversity and exploring opportunities for the valorization of hop biomass.

## Results and Discussion

### Profiling of Phyllobilins in Senescent Hop Leaves

Senescent hop leaves were collected in Hüll, Germany, after the harvest for beer-brewing was completed, in late autumn in 2019–2021. The methanolic extract of a sample of ∼ 5 g of senescent, yellowish hop leaves was examined by analytical HPLC to profile the presence of PBs. PBs were assigned by their online UV/vis spectra from a diode array detector along with LC-MS analysis to identify typical fragmentation patterns of linear tetrapyrroles.^19^ The relative content of yellow PBs was assessed by their HPLC peak areas relative to the sum of peak areas at 420 nm. In this manner (Figure 2, S1a and S1b), six signals from DPleBs and DPxBs were identified. Two particularly stood out, since the analytical data suggested a modification pattern previously unknown for PBs.

**Figure 2:**
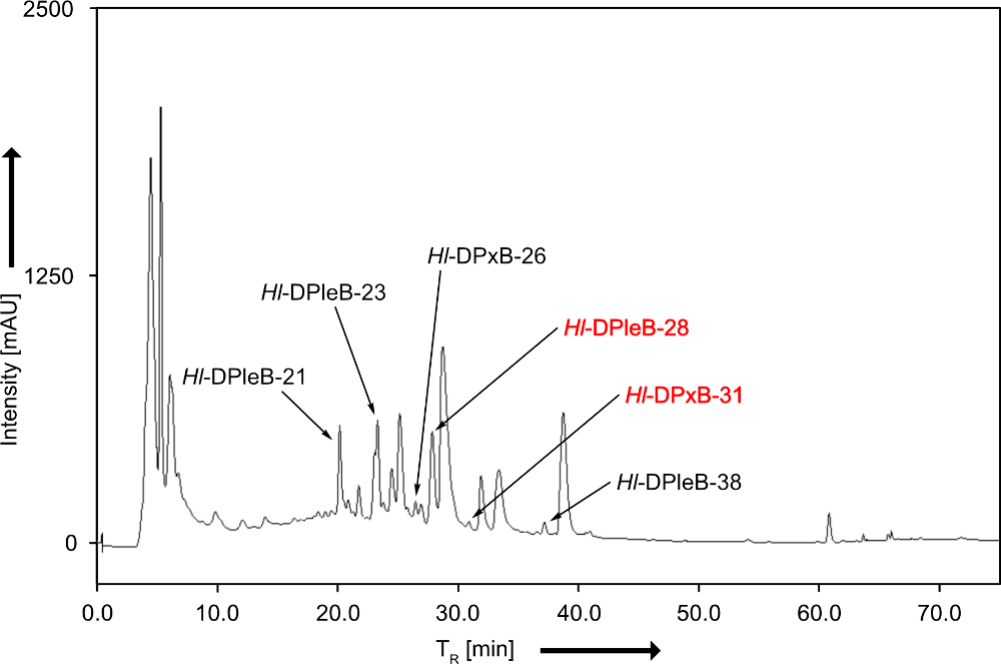
LCMS-analysis of an extract of senescent leaves of *Humulus lupulus* using analytical HPLC (detection at 250 nm). *Hl*-DPleB-28 and *Hl*-DPxB-31 are highlighted in red. For UV/vis and MS spectra, see the Supporting Information (Figures S1a and S1b).

PBs are usually labeled according to their botanical source and subclass characteristics, designating them as *Hl*-PBs for compounds from *Humulus lupulus*. The presumably new *Hl*-PBs were found by their UV-spectra to be type-II PBs, one DPleB and one DPxB. DPxBs have so far only been scarcely identified in plants; relative quantification by HPLC revealed that the relative abundance of *Hl*-DPxB was highest in yellow hop leaves (Figure 3). Specifically, *Hl*-DPxB constituted approximately 40% of all peak areas at 420 nm, indicating its significant contribution to the yellow coloration of hop leaves in autumn. In contrast, in green and already brown leaves, *Hl*-DPxB accounted for 4% and 11% of peak areas, respectively. These results are in line with the visual appearance of the leaves and the overall known increase of PBs during maturing and senescence of plant material,^18^ affecting the overall composition of hop leaves. The formation of *Hl*-DPxB, as indicated by the relative peak areas at 420 nm, therefore contributes substantially for the yellow coloration observed in the leaves (Figure 3), as was already indicated by a previous study on *Cercidiphyllum japonicum*, in which a type-I PxB accounted for approximately 5–10% of coloration of senescent leaves.^11^

**Figure 3:**
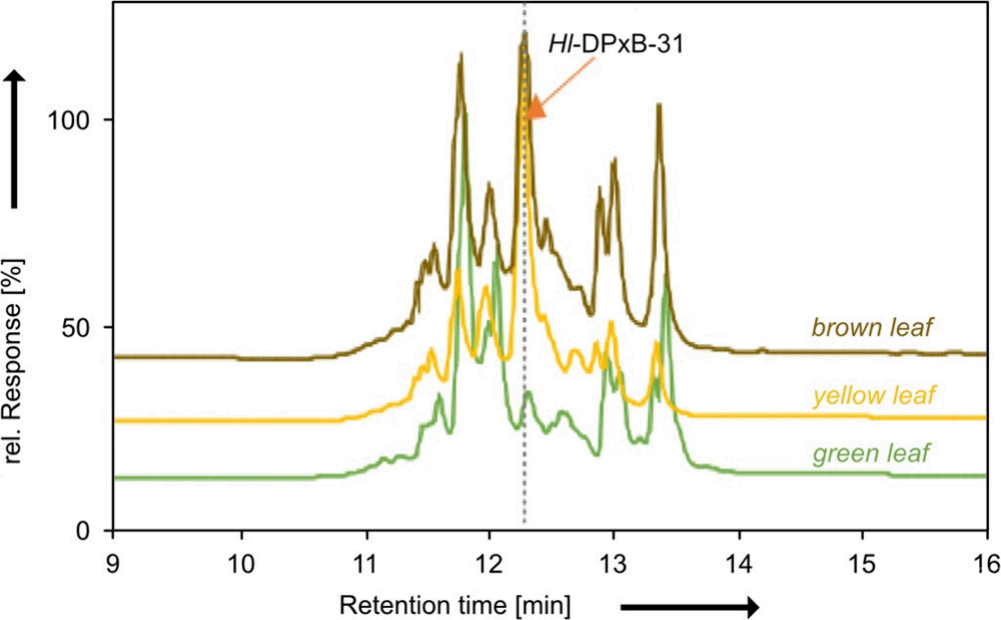
Chromatograms of the HPLC analysis (detection at 420 nm) of methanolic *Humulus lupulus* leaf extracts made from leaves of different colors. Relative peak areas of DPxB were highest for yellow leaves (36.7%), followed by brownish leaves (15.1%), and least in green leaves (3.3%).

### Preparative Isolation of Phyllobilins from Senescent Hop Leaves

Isolation of PBs is inherently challenging due to (1) the complexity of the mixture, and (2) sensitivity of tetrapyrromethanes to light and air. Here, the isolation of hop PBs proved to be even more difficult than the isolation of PBs from other sources due to an unsuppressible isomerization, suggesting unique structural features. Regardless, preparative purification of 107 g of wet senescent leaves gave 0.24 mg (0.37 μmol) of *Hl*-DPleB-28 and 0.80 mg (1.24 μmol) of *Hl*-DPxB-31 (see Supporting Information for more details). The structure elucidation is described next.

### Structural Characterization of PBs from Hop Leaves

The crystallization of these types of natural phyllobilins has not yet been achieved (other than for a semisynthetic derivative^20^). Still, spectroscopic characterization is well established for this class of compounds.^8^ The UV/vis spectra showed absorption near 254 nm for *Hl*-DPleB-28 and near 420 nm for *Hl*-DPxB-31. Thus, the spectra showed similarities to other previously described DPleBs and DPxBs (Supporting Information and Figures S4 and S5).^21,22^ The ECD spectra of *Hl*-DPleB-28 and *Hl*-DPxB-31 showed similarities with previously known ECD spectra of *Hordeum vulgare* or *Cercidiphyllum japonicum* ^23^ and indicated well-observed configurations at C10 (Figures S5 and S6).^24^ The molecular formulas of *Hl*-DPleB-28 and *Hl*-DPxB-31 were derived from the high-resolution mass spectra and determined as C_34_H_40_O_9_N_4_ and C_34_H_38_O_9_N_4_, respectively (Figures S1a and S1b). As shown in Figure S8, ESI-MS analysis of *Hl*-DPleB-28 in positive ion mode revealed a [M + H]^+^-ion signal at *m*/*z* = 649.28. Fragment ions at *m*/*z* = 617.26 and 510.22 indicated the loss of MeOH (from a methyl ester functionality), as well as of C_7_H_9_NO_2_ (ring D). However, ESI-MS analysis of *Hl*-DPxB-31 in positive ion mode showed a [M + H]^+^-ion signal at *m*/*z* = 647.27, and the absence of the fragment ion of about 508.20, as is observable due to the loss of ring D in DPleB chromophores,^19^ indicated a double bond at C15 (Figure S9). Homonuclear and heteronuclear NMR spectra provided signal assignments for *Hl*-DPleB-28 and *Hl*-DPxB-31 and were used to determine their molecular constitutions (see Figures 4 and 5 as well as Table S1). ^1^H NMR spectra of solutions of *Hl*-DPleB-28 and *Hl*-DPxB-31 in CD_3_OD (Figures S10 and S11) showed characteristic signals for a vinyl group around 6 ppm, a signal at 3.77 ppm, suggesting a methyl ester functionality and typical signals for the methyl groups around 2 ppm. However, there were signals for only three methyl groups instead of four, indicating a modification at one of them. New AB systems were identified at 4.64/4.44 ppm (*Hl*-DPleB-28) and 4.63 ppm (*Hl*-DPxB-31), which showed NOE correlations in a ^1^H,^1^H-ROESY spectrum with the ABX system of the vinyl group at ring D (6.09/5.43 and 6.56 ppm for the PleB, and 6.28/5.42 and 6.71 ppm for the PxB, respectively), and with the singlet at 1.98 ppm (*Hl*-DPleB-28) and 2.17 ppm (*Hl*-DpxB-31), respectively, from the methyl group of ring C, and was assigned to a hydroxymethyl group at position C17^1^. The newly identified *Hl*-DPleB-28 was therefore shown to be a 17-hydroxymethyl-DPleB, and the *Hl*-DPxB-31 a 17-hydroxymethyl-DPxB, as shown in Figures 4 and 5.

**Figure 4:**
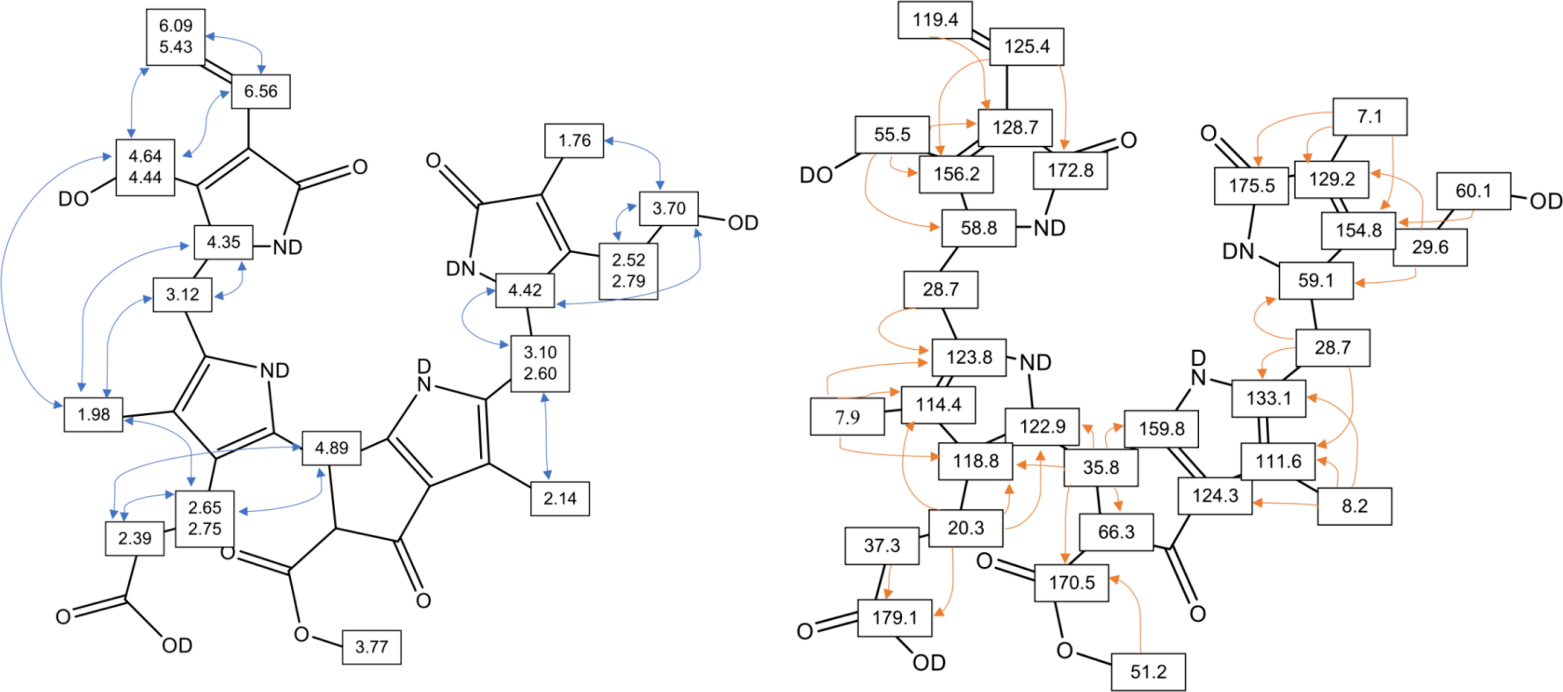
Left: Heteronuclear single- and multiple-bond correlations (from ^1^H,^1^H–COSY and ^1^H,^1^H-ROESY-spectra, respectively) and assignments of ^1^H-signals of *H* *l*-DPleB-28. Right: Heteronuclear single- and multiple-bond correlations (from ^1^H, ^13^C-HSQC and ^1^H, ^13^C-HMBC-spectra, respectively) and assignments of ^13^C-signals of *Hl*-DPleB-28.

**Figure 5:**
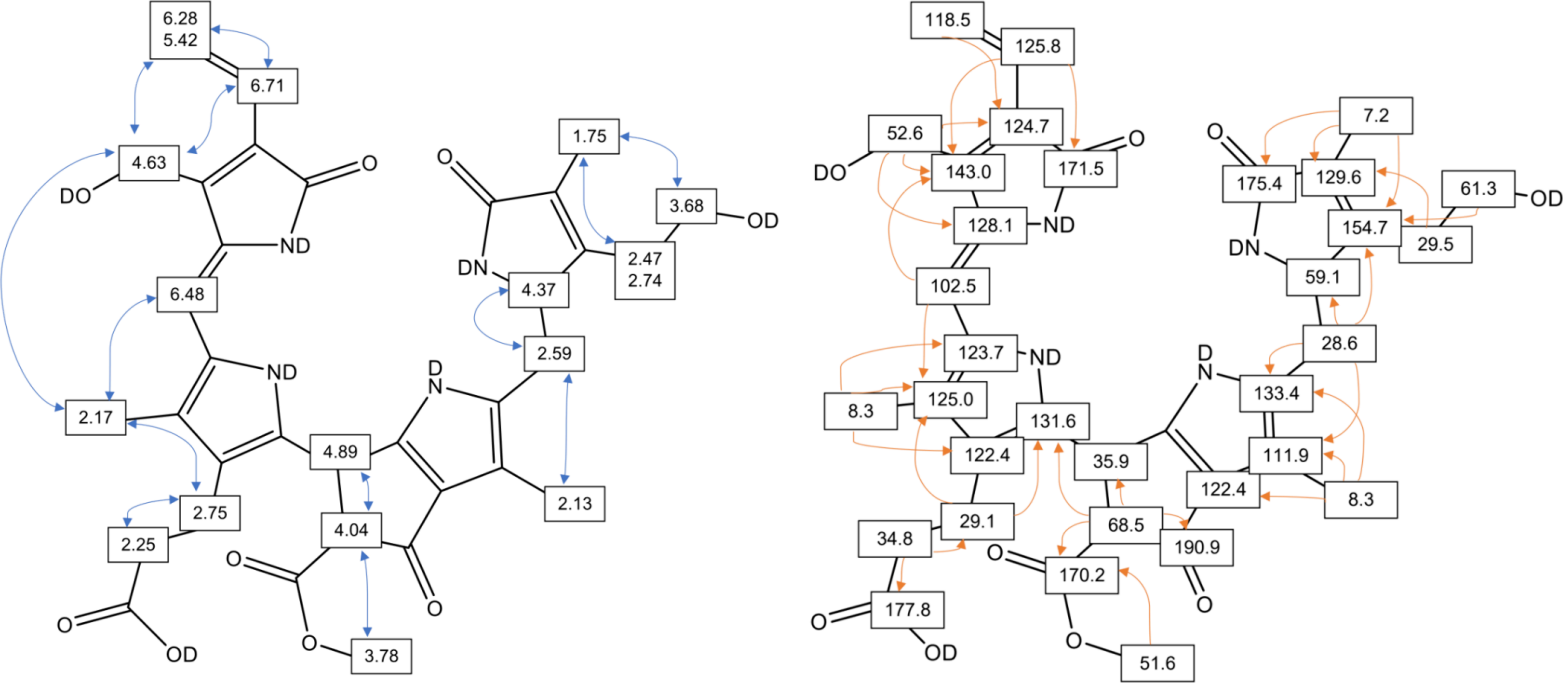
Left: Heteronuclear single- and multiple-bond correlations (from ^1^H,^1^H–COSY and ^1^H,^1^H-ROESY-spectra, respectively) and assignments of ^1^H-signals of *Hl*-DPxB-31. Right: Heteronuclear single- and multiple-bond correlations (from ^1^H, ^13^C-HSQC and ^1^H, ^13^C-HMBC-spectra, respectively) and assignments of ^13^C-signals of *Hl*-DPxB-31.

Furthermore, the ^1^H NMR spectrum of *Hl*-DPleB-28 (Figure S10) also showed an additional, slightly shifted set of signals, suggesting the presence of two isomers *Hl*-DPleB-28 and *Hl*-DPleB-28a. Indeed, a mixture of two isomers was confirmed by isocratic HPLC analyses revealing two fractions with identical UV/vis spectra (Figure S2) as well as by the comparison of the ^1^H NMR data (Table S2). The spectrometric and spectroscopic data of the two isomers are identical except for small differences in the ^1^H NMR spectra. Slight deviations of the chemical proton shifts, in particular at the positions C10, C12^1^ and C12^2^ as well as C17^1^, indicate an isomerization due to the acidic hydrogen at the C8^2^ position, a well characterized phenomenon known for phyllobilins.^23^ As depicted in Figures 4 and 5, ^1^H,^1^H homonuclear correlations from ROESY spectra and ^1^H,^13^C-heteronuclear correlations from HMBC spectra finally revealed a previously unknown additional hydroxylation at ring D.

### Hydroxylation Pathway(s)

While hydroxylation at the terminal carbon C3^2^ has been described as the first modification of a primary fluorescent chlorophyll catabolite (primary phyllolumibilin (pPluB), formerly termed pFCC) to form 3^2^-hydroxy-pPluBs within the senescing chloroplast,^25^ the presence of a hydroxymethyl group at C17^1^ of a nongreen chlorophyll degradation product from a higher plant is unprecedented; one hypothesis could be that this structure arises from a variation of the chlorophyll catabolic pathway in *Humulus lupulus*. The hydroxylation presumably occurs before, during, or after transport to the cytosol and during modification of *p*PluBs to modified PluBs (*m*PluBs, Figure 6); another mechanism, however, might be possible – the discovery of the 2^1^ hydroxylated phyllobilins adds another unresolved question to the already so many on those natural products. The additional hydroxylation might be the cause for the observed isomerization, which is uncommon for PBs. Indeed, PBs are known to occur as isomers in a ratio of about 9/1, due to an inversion at C 8^2^ caused by an acidic proton.^23^ The closest stereocenter, which could be affected by the hydroxylation, is C16. The absolute configuration of this stereocenter was found to be plant specific and enzyme dependent.^27^ Although the absolute configuration is still unknown, no epimerization at this stereocenter was found up to now. Elucidating the absolute configuration is the planned subject of future studies on PBs, which include working toward the total synthesis of phyllobilins.^28^

**Figure 6:**
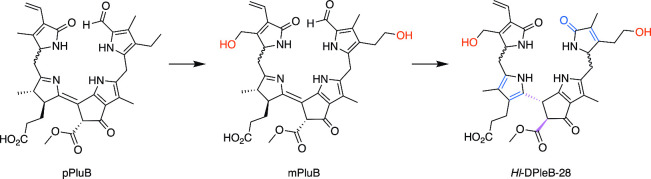
Hypothetical formation of *Hl*-DPleB-28 in common hop plants. The hydroxylation at the “western” hemisphere is presumably introduced at the stage of the primary fluorescent chlorophyll catabolite (phyllolumibilin, *p*PluB, formerly known as *p*FCC) yielding a modified PluB (*m*PluB), as is the hydroxylation of the ethyl side chain at ring A.^25^

### Comparison of Known Phyllobilins

The new PBs display a previously unobserved hydroxylation in the ‘western’ hemisphere of the tetrapyrrolic molecule at a methyl group; by contrast, phyllobilin modifications heretofore have typically been observed at the vinyl group at ring D or the ethyl side chain at ring A. There is one example from *Arabidopsis thaliana*, in which a type-I PB with a hydroxylation at the methyl group of ring A was identified.^29^ This hydroxylation was unusual, since PBs all appear to originate from chlorophyll *a* rather than from chlorophyll *b*, which was rationalized by the discovery of the chlorophyll *b* to *a* reduction system that was found to precede the degradation pathway of chlorophyll. However, this reduction proceeds via hydroxymethyl-Chl *a*,^30^ and the metabolite found in *Arabidopsis* could therefore stem from an incomplete reduction. Also in *Arabidopsis thaliana*, hydroxymethylated DPleBs have been characterized that do not follow the ‘usual’ modification patterns.^21^ Such modifications have been observed in a previous study using *Arabidopsis* mutants.^31^ In the metabolite from hops, the discovery of the hydroxylation of a methyl group at the tetrapyrrole core could possibly indicate an unprecedented still unidentified modifying enzyme, or, even more remarkably, a new catabolic pathway. While the insertion of the hydroxyl group at this position is rather novel, it warrants mention that chlorophyll *f*, a far-red absorbing chlorophyll species, contains a formyl group flanking the vinyl group (ring A in the macrocycle, ring D in the PB).^32^ Whether there is any similarity in enzymic processes affording the two species remains to be determined.

### Antioxidant Properties

Prior studies showed potent antioxidative effects of a DPleB^33^ and a DPxB^34^ with modification patterns that differ from the ones found in metabolites of the common hops. Here, the two new PBs identified from hops were tested for their antioxidant activity *in vitro* and *in cellulo*. For the *in vitro* assay, a FRAP assay^35^ was performed, using Trolox as a standard. *Hl*-DPxB-31 proved to be a potent antioxidant in the *in vitro* FRAP assay. The antioxidative effect was comparable to Trolox, a vitamin E derivative. In contrast, *Hl*-DPleB-28 exhibited a significantly lower antioxidative effect compared to Trolox, which may be due to the poor solubility of *Hl*-DPleB-28 in 75% EtOH (Figure 7). To investigate the antioxidative activity of the newly identified compounds, an *in cellulo* assay was performed with the conversion of H_2_DCF to DCF by hydrogen peroxide as read-out.^36^ *Hl*-DPxB-31 and *Hl*-DPleB-28 were tested, as well as quercetin, a known flavonoid constituent of hops.^31^ All tested compounds showed ROS scavenging activity. *Hl*-DPxB-31 and *Hl*-DPleB-28 scavenged ROS in cells in a manner comparable to that of Trolox and quercetin. As a control, the influence on cell viability was assessed for the tested compounds. Trolox, *Hl*-DPxB-31, and *Hl*-DPleB-28 had no effect on cell viability at these concentrations. Quercetin elicited a moderate reduction in cell viability (Figure 8). An additional way to mediate cellular protection against oxidative and electrophilic stress can be provided by activation of signaling processes such as the factor–erythroid 2-related factor 2 (Nrf-2)/ antioxidant response element (ARE) pathway.^37^ Nrf-2 promotes the transcription of genes that are involved in cytoprotection and phase II detoxification such as NAD­(P)­H:quinone oxidoreductase 1 (NQO1), glutathione-*S*-transferases (GST), or heme oxygenase-1 (HMOX1). By upregulating the expression of these enzymes, the cellular antioxidative capacity is enhanced.^37–39^ To analyze the transcriptional activation of ARE-driven reporter gene expression, the CellSensor® ARE-bla HepG2 cell system was used. As shown in Figure S14, ARE-driven β-lactamase expression was stimulated 2.2 ± 0.3-fold after treatment of CellSensor® ARE-bla HepG2 cells with 50 μM quercetin for 18 h. The *Hl*-DPxB was tested in a concentration range of 6.25 to 200 μM; however, no dose-dependent significant activation of the reporter system was observed. Still, a slight upregulation of reporter gene expression can be observed starting from 25 μM *Hl*-DPxB. Our exploratory results on antioxidative activities on hops therefore confirm previous results on the antioxidative potency of structurally related phyllobilins from other plant sources: Antioxidative testing of a DPleB from *Vitis vinifera* revealed a potent effect *in vitro* and *in cellulo*, and the effect turned out to be stronger compared to the type-I equivalent of the tested DPleB.^33^ A DPxB isolated from Savoy cabbage showed a more potent antioxidative activity than bilirubin in a FRAP and DPPH free radical scavenging assay.^34^ Hence, while type-II PBs are antioxidative natural products that have yet to be considered in phytochemical analyses, *Hl*-DPxB as well shows antioxidative potential in exploratory *in vitro* and cellular assays. Such results, which need to be further addressed, could represent an incentive for upcycling of biomass from hop leaves.

**Figure 7:**
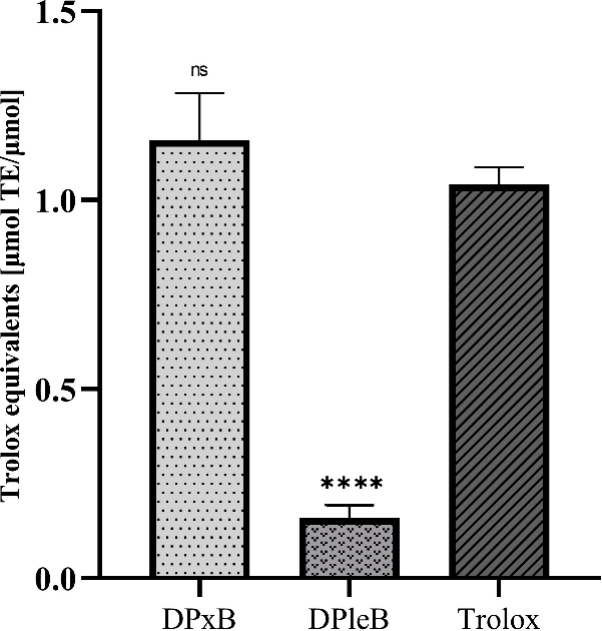
*In vitro* antioxidative effects of hop type-II PBs tested via a FRAP Assay. Values represent mean ± standard deviations of three independent experiments (ns = not significant, *p* < 0.05).

**Figure 8:**
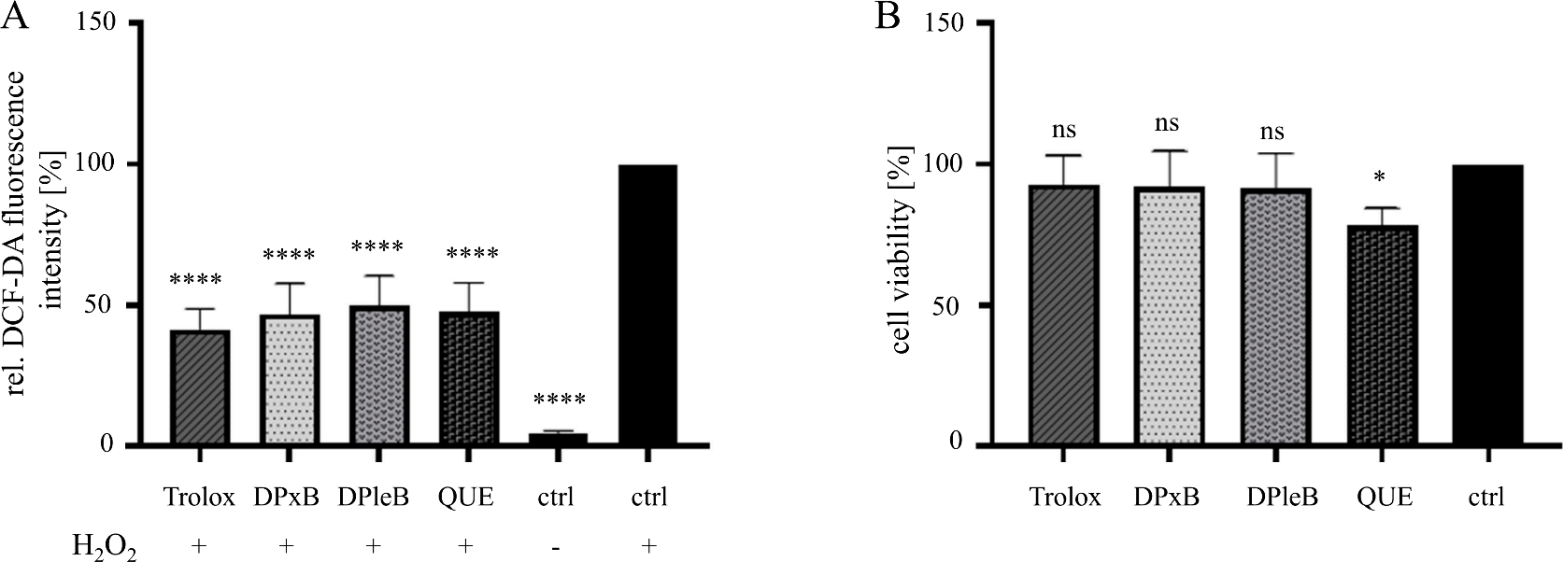
Type-II PBs scavenge ROS in HeLa cells (A) without affecting cell viability (B). (A) Cells were treated for 24 h with compound (10 μM, Trolox 1 mM), then stimulated with dye (30 min) and H_2_O_2_ (30 min). Generation of ROS was measured as conversion of dye H_2_DCF to the fluorophore DCF, and radical scavenging activity represents the prevention of DCF formation. (B) *Hl*-DPxB-31, *Hl*-DPleB-28 and Trolox do not affect cell viability at 10 μM or 1 mM for Trolox, respectively. Quercetin (QUE) influences cell viability significantly at a concentration of 10 μM. Cell viability was assessed as a control experiment by a crystal violet staining after 24 h stimulation of compounds in the indicated concentrations. Values represent mean ± standard deviation of four independent experiments (ctrl = control, ns = not significant, *p* < 0.05).

### Bitterness Assay

Since hops is appreciated for its bitter compounds, the question arose whether the newly identified structures could contribute to the bitter sensation of hops. The prediction tool VirtualMultitaste^40^ was used to test for the bitterness of phyllobilins in hops, with comparisons made to (1) structural representatives of phyllobilins; (2) humulone and lupulone, known bitter substances in hops; and (3) strychnine and amarogentin, which are among the most recognized natural bitter substances. The prediction tools suggested a bitter taste for *Hl*-PleB and *Hl*-DPxB with probabilities of 66 and 60%, respectively (Figure 9 and Figure S15). In comparison, a PleB and a PxB isolated from *Cercidiphyllum japonicum*, which represent the most common and basic phyllobilin structures, showed a slightly lower probability of around 55%. As expected, humulone and lupulone gave high bitterness probabilities of 89% and 94%, respectively, while amarogentin showed a probability of 92%. Interestingly, the bitterness of strychnine, which has recently been shown to bind directly to the human bitter taste receptor 46,^41^ was predicted with a lower probability of 75%. While the results are computational in nature and require experimental verification, a working hypothesis is that the newly identified hydroxymethyl structural motif of phyllobilins in hops may enhance the chemical attributes associated with bitterness.

**Figure 9:**
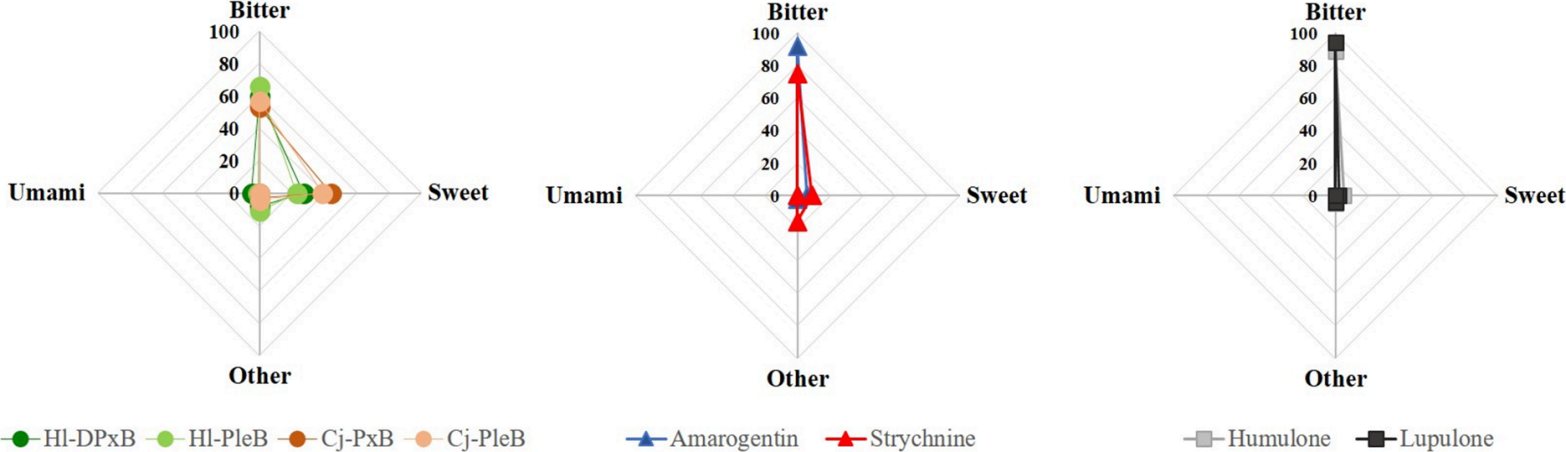
Predicted bitterness of phyllobilins isolated from hops, structural representatives of the phyllobilin natural product class (*Cj*-PleB and *Cj*-PxB, left), known bitter compounds from hops (humulone and lupulone, right), and the well-known bitter compounds amarogentin and strychnine (middle). Bitterness prediction was performed using VirtualMultitaste, a multiclass prediction tool designed to classify compounds as bitter, sweet, or umami (https://virtuous.isi.gr/#/virtuous-multitaste).^40^

A preliminary assessment of the effects of *Hl*-PleB and *Hl*-DPxB on the mRNA expression of selected human bitter taste receptors revealed no significant impact on receptor expression levels; but neither did amarogentin and strychnine (Figure S16, Supporting Information). It is now known that ‘bitter taste’ is a complex taste modality due to the plethora of bitter molecules and the multiple bitter taste receptors.^42^ Indeed, the number of molecules classified as bitter is >2000.^42^ While taste alone is an important sensation, bitter-taste receptors are known to sense diverse molecules in nongustatory physiological regulation.^43^ Virtual tools for bitterness are very useful for predictive assessments. The pharmacological investigation into the potential role of phyllobilins as bitter compounds—and their contribution to the flavor and aroma of hops—will be the focus of future studies.

The findings reported here not only enrich our understanding of PBs as bioactive compounds, but also the diversity in plants and the knowledge of the pathway underlying the degradation of chlorophyll. The biochemical formation of hydroxymethylated dioxophyllobilins in hop plants still waits to be characterized. Indeed, the identification of potentially bitter phyllobilins in senescent hop leaves not only adds to the chemical diversity of hops,^44^ yet also could lead to a more sustainable use of this important crop, adding to the already ongoing incentives of recovery of bioactive compounds from hop leaves for the valorization of agricultural byproducts.^45^

## Experimental Section

### Materials

Ethanol (EtOH) was purchased from VWR International GmbH (Ismaning, Germany), ultrapure water (18 MΩ.cm^–1^) from a Millipore S.A.S Milli-Q Academic system (18,2 MΩ cm^–1^, Molsheim, France) and hydrogen peroxide (30%) was from Bernd Kraft (Duisburg, Germany). 2,4,6-Tri­(2-pyridyl)-s-triazine (TPTZ), iron­(III) chloride (FeCl_3_), quercetin were from Merck (Darmstadt, Germany). Trolox was from Enzo Life Sciences GmbH (Lörrach, Germany), 2′,7′-dichlorodihydrofluoroscein diacetate (H_2_DCF-DA) were from Thermo Fisher (Waltham, MA, USA) and DMSO was from Carl Roth (Karlsruhe, Germany). DMEM medium was obtained from PAN-Biotech (Aidenbach, Germany); Fetal calf serum (FCS) was from PAA Laboratories GmbH (Pasching, Austria).

### Plant Material

Senescent hop leaves were collected and provided by the hop research center Hüll, Germany (Bayerische Landesanstalt für Landwirtschaft (LfL), Hopfenforschungszentrum Hüll, Wolnzach, Germany) in the years 2019, 2020, and 2021, specifically on October 19, 2019, September 29, 2020, and September 29, 2021. Hop plants are typically harvested when they reach their peak brewing value, not necessarily when they are physiologically mature. In the Hallertau region, the harvest period varies depending on weather conditions and hop variety, generally occurring between the end of August and September 20–25.

For this study, leaves were collected after the regular hop harvest had been completed. The samples were taken from hop seedlings that did not exhibit sufficient positive characteristics for harvesting, as only about 3% of planted seedlings are actually harvested. These seedlings are shredded after the regular harvest. Therefore, the samples investigated in this study represent a mixture of various genotypes, providing an average representation of the ″Hüller hop genetics.″

### Analysis of PBs by HPLC

About 5 g of senescent, yellowish hop leaves were ground in a mortar, extracted with 1 mL of MeOH, diluted with 4 mL of water, centrifuged at 13400 rpm, and applied to analytical HPLC. Relative quantification of DPxB: A piece of approximately 1 cm^2^ each of four different leaves of *Humulus lupulus* was cut out and ground with 200 μL of MeOH in a mortar, diluted 1:11 with potassium phosphate buffer 100 mM pH 7 (20/80), and centrifuged. A 100 μL portion of the extract was analyzed by analytical HPLC. PxBs were assigned by their online UV/vis spectra from a diode array detector, and their relative content was assessed by their HPLC peak areas relative to the sum of peak areas at 420 nm.

### HPLC

Dionex Ultimate 3000 HPLC system, ultimate 3000 pump, ultimate 3000 diode array detector and RF2000 fluorescence detector, 200 μL or 2 mL injection loop. a) Analytical HPLC chemoprofiling: Phenomenex HyperClone ODS 5 μm 250 × 4.6 mm i.d. column at RT connected to a Phenomenex ODS 4 mm × 3 mm i.d. precolumn; flow rate was 0.5 mL min^–1^. Solvent A: water with 4 mM ammonium acetate, solvent B: MeOH with 4 mM ammonium acetate, solvent composition (A/B) 0–5 min: 80/20; 5–55 min: 80/20 to 30/70; 55–60 min: 30/70 to 0/100; 60–70 min: 0/100; 70–75 min: 0/100 to 80/20. Data were collected and processed with Chromeleon. b) Analytical HPLC relative quantification: Agilent 1260 Infinity II LC system with a 1260 Infinity Degasser, a 1100 Series quaternary pump, and a 1100 Series diode array detector connected to an Agilent Poroshell column 120EC-C18 4 μm 4.6 × 150 mm with Phenomenex ODS 4 × 3 mm i.d. precolumn; flow rate was 0.5 mL min^–1^, injection volume 100 μL. Solvent A: Ammonium acetate buffer 10 mM pH = 7, solvent B: ACN, solvent composition (A/B) 0–2 min: 95/5; 2–17 min 95/5 to 0/100; 17–20 min 0/100, 20–22 min 0/100 to 95/5. Data were processed with Agilent OpenLab CDS. c) Preparative HPLC: Thermo HYPERSIL ODS 5 μm 250 × 21.2 mm i.d. column at RT, flow rate was 5.0 mL min^–1^. Solvent A: 4 mM ammonium acetate in H_2_O, solvent B: MeOH with 4 mM ammonium acetate, solvent composition (A/B) 0–5 min: 80/20; 5–34 min: 80/20 to 50/50; 34–36 min: 50/50 to 0/100; 36–40 min: 0/100; 40–42 min: 0/100 to 80/20. Data were collected and processed with Chromeleon.

### Spectroscopy

All plant materials (yellow and green leaves) were collected from different hop plants *(Humulus lupulus)* and analyzed freshly, or stored at −80 °C for further use. UV/vis: Agilent Technologies Cary 60 UV–vis λ_max_ (nm)/(log *ε*). ^1^H- and ^13^C NMR: 700 MHz Avance 4 Neo, 600 MHz Avance II+ spectrometer; ^1^H NMR: 400 MHz Bruker Avance 4 Neo spectrometer; residual solvent peaks (CD_3_OD: δ_H_ = 3.31 ppm; ^13^CD_3_OD: δ_C_ = 49.0 ppm).

### Mass Spectrometry and LC-MS

A Dionex Ultimate 3000 HPLC system (see paragraph HPLC) was coupled to a ThermoScientific QExactive mass spectrometer, equipped with an ESI source (positive-ion mode, spray voltage 3.2 kV). MS and MS^n^ Data were processed with Xcalibur and mMass.^46^

### Isolation and Structure Elucidation

From 107 g of senescent leaves (wet weight), 0.24 mg (0.37 μmol) of *Hl*-DPleB-28 and 0.80 mg (1.24 μmol) of *Hl*-DPxB-31 were isolated, which showed the following spectroanalytical data (see Supporting Information for further details). *Hl*-DPleB-28: UV/vis (MeOH, c = 5.2 × 10^–5^_M_): λ_max_ (ε_rel_) = 214 (1.0), 240 (0.72); MS (ESI^+^): *m*/*z* (%): 687.24 (24, [M+K]^+^); 671.27 (15, [M + Na]^+^), 651.30 (06), 650.29 (36), 649.29 (100, [M + H]^+^, C_34_H_41_O_9_N_4_^+^). *Hl*-DPxB-31: UV/vis (MeOH, c = 2.4 × 10^–5^_M_): λ_max_ (ε_rel_) = 218 (0.88), 420 (1.0); MS (ESI^+^): *m*/*z* (%): 685.23 (06, [M+K]^+^); 669.23 (12, [M + Na]^+^), 649.28 (06), 648.27 (35), 647.27 (100, [M + H]^+^, C_34_H_39_O_9_N_4_^+^).

### Sample Preparation and Ferric Reducing Antioxidant Potential (FRAP) Assay

DMSO stocks of *Hl*-DPxB-31, *Hl*-DPleB-28, and quercetin were prepared from lyophilized samples and stored at −20 °C. The FRAP assay was performed according to the protocol of Benzie et al.^35^ with minor modifications. 100 μM of compounds and different concentrations of Trolox were prepared in 75% EtOH and incubated with the FRAP solution for 5 min at 37 °C in a 96 well plate. The FRAP reagent was freshly prepared by adding 300 mM acetate buffer pH = 3.6, 10 mM TPTZ (2,4,6-Tri­(2-pyridyl)-s-triazine) in 40 mM HCl and 20 mM iron­(III)­chloride in a 10:1:1 ratio. The assay utilizes the ability of antioxidants to reduce the Fe^3+^-(TPTZ)_2_-complex to Fe^2+^-(TPTZ)_2_, of which the absorbance can be measured at 593 nm. The antioxidant power of the compounds was calculated as Trolox equivalents by using a Trolox calibration curve.

### Cell Culture

HeLa cells were obtained from the *Deutsche Sammlung von Mikroorganismen and Zellkulturen* (DSMZ; Braunschweig, Germany) and cultured in DMEM medium supplemented with 10% fetal calf serum (FCS). Cells were cultured at 37 °C under 5% CO_2_ atmosphere and divided in a 1:10 ratio every 3 to 4 days.

### Intercellular Reactive Oxygen Species (ROS) Assay

The intercellular ROS assay was performed according to a published protocol.^36^ In brief, 1 × 10^5^ cells/mL of HeLa cells were seeded in 96 well plates. After 24 h of incubation, cells were treated with *Hl*-DPxB-31 (10 μM), *Hl*-DPleB-28 (10 μM), quercetin (10 μM), Trolox (1 mM), or a DMSO control, and incubated again for 24 h. After the medium was removed, the cells were treated with H_2_DCF-DA (10 μM) and incubated for 30 min. Then, the cells were washed with PBS and incubated with hydrogen peroxide (1 mM) for 30 min. The generation of ROS was measured by the intercellular oxidation of H_2_DCF to highly fluorescent 2′, 7′-dichlorofluorescein (DCF) and detected using a Tecan SectraFluor plus microplate reader (excitation wavelength 485 nm; emission wavelength 530 nm). Data were normalized to the hydrogen peroxide treated DMSO control. The effect of compound treatment on cell viability was tested by crystal violet staining, measured with a spectrophotometer at 590 nm. The number of viable cells was normalized to a DMSO control.

## Supplementary Material



## Notes

Chemicals and instruments used in this study; collection, isolation, and spectroanalytical data of *Hl-*DPleB-28 and *Hl*-DPxB-31 (including UV/vis and ECD spectra, MS^2^ analyses, ^1^H NMR spectra, and ^13^C- and ^1^H NMR chemical shift values (CD_3_OD, 25°) and signal assignments of *Hl*-DPleB-28 (600 MHz (^1^H)/150 MHz (^13^C)) and *Hl*-DPxB-31 (700 MHz (^1^H)/175 MHz (^13^C)); online UV/vis and MS spectra of hops phyllobilins; isocratic HPLC analysis of the *Hl*-DPleB-28 fraction; ^1^H NMR chemical shift values of the two isomers of *Hl*-DPleB-28 (CD_3_OD, 25 °C) extracted from heteronuclear ^1^H, ^13^C-HSQC and ^1^H, ^13^C-HMBC-spectra (600 MHz (^1^H)/150 MHz); reagents and chemicals for antioxidative testing; Nrf-2/ARE reporter gene assay; bitterness assessment (reagents and chemicals, multitaste prediction, mRNA expression levels of human bitter taste receptors); references (PDF)
