# Supplementary material for: Characterization of Phyllobilins in Hops: Antioxidant and Potentially Bitter Senescence-Related Metabolites
Source: J Agric Food Chem. 2025 Jul 7;73(28):17637–45. doi: 10.1021/acs.jafc.5c03549 (PMC12272678; doi:10.1021/acs.jafc.5c03549)
Supplement: Supplementary file 1 [file jf5c03549_si_001.pdf]

Characterization of Phyllobilins in Hops: Antioxidant and Potentially Bitter Senescence-Related Metabolites

Supporting Information

Christian Nadegger,[a,b] Patricia Frei,[c] Christian A. Elvert,[b, d] Cornelia A. Karg,[b, d] Johanna M. Gostner,[e] Jonathan S. Lindsey,[f] Christoph R. Kreutz,[a,b] Stefan Schwaiger,[b, d] Thomas Müller,[a,b]* and Simone Moser [b, d]*

a Institute of Organic Chemistry, University of Innsbruck
Innrain 80/82, A-6020 Innsbruck (Austria)

b Center for Molecular Biosciences, University of Innsbruck
Innrain 80/82, A-6020 Innsbruck (Austria)

c Department of Pharmacy, University of Munich
Butenandtstraße 5-13, D-81377 Munich (Germany)

d Department of Pharmacognosy, Institute of Pharmacy, University of Innsbruck,
Innrain 80/82, A-6020 Innsbruck (Austria)

e Institute of Medical Biochemistry, Medical University of Innsbruck, #
Innrain 80/82, A-6020 Innsbruck, Austria)

f Department of Chemistry, North Carolina State University,
Raleigh, NC 27695 (USA)

* To whom correspondence should be addressed: thomas.mueller@uibk.ac.at, simone.moser@uibk.ac.at

Chemicals: HPLC-grade methanol (MeOH), *n*-hexane and ethyl acetate were purchased from Merck (Darmstadt), Acros Organics, and Sigma Aldrich. Ammonium acetate puriss. p.a. was from Fluka (CH-Buchs) and ultrapure water (18 $M\Omega cm^{-1}$) was from a Millipore apparatus. Sep-Pak-Cartridges (5 g) were from Waters Associates.

Ultraviolet/visible (UV/Vis) spectroscopy: Agilent Technologies (type: Cary 60 UV-Vis), in MeOH; λ_{max} [nm] (ε_{rel}); 10 x 10 mm UV-cells (quartz)

Electronic Circular dichroism spectrometry (ECD): JASCO J-1500 λ_{max} and λ_{min} (nm)/$\delta\varepsilon$.

^{1}H- and ^{13}C-Nuclear Magnetic Resonance Spectroscopy (NMR): 700 MHz Avance 4 Neo, 600 MHz Avance II+ spectrometer, and 400 MHz Bruker Avance 4 Neo spectrometer, δ($C^{1}HD_2OD$) = 3.31 ppm[1], and δ($^{13}CD_3OD$) = 49.0 ppm[1], ^{1}H- and ^{13}C- signal assignments from homonuclear (^{1}H,^{1}H-COSY and ^{1}H,^{1}H-ROESY) and heteronuclear (^{1}H,^{13}C-HSQC and ^{1}H,^{13}C-HMBC) experiments.[2]

Mass spectrometry (MS) and Tandem mass spectrometry (MS^n)[3]: ThermoScientific QExactive, equipped with an ESI source (positive-ion mode, spray voltage 3.2 kV, solvent: MeOH/4 mM ammonium acetate). Data collection and processing with Xcalibur 4.1 software.

Collection, isolation, and structure elucidation of *Hl*-DPleB-28 and *Hl*-DPxB-31.

Senescent, yellowish hop leaves (about 10 g wet weight) were ground in a mortar, extracted with MeOH, and concentrated under vacuum. The methanolic supernatant was then diluted with water and applied to a SepPak-cartridge (5 g). The crude product was eluted with using 2 ml of MeOH, dried under vacuum, and re-dissolved in 300 µl of MeOH. After the addition of 400 µl of *n*-hexane and 200 µl of ethyl acetate, three phases appeared. The middle yellow phase was collected, and the solvent was removed using a rotary evaporator and high vacuum. Thereafter 400 µl of MeOH and 1600 µl of water were added. The obtained 2 ml mixture was applied to preparative HPLC and the corresponding fractions of *Hl*-DPleB-28 and *Hl*-DPxB-31 were collected. This procedure was repeated nine times. After the repetitions, the corresponding collected fractions were combined and dried under high vacuum. For further purification, the fractions were subjected to analytical HPLC, desalted by using a 5 g Sep-Pak-cartridge, and dried under vacuum to yield 0.24 mg (0.37 µmol) of *Hl*-DPleB-28 and 0.80 mg (1.24 µmol) of *Hl*-DPxB-31.

Spectroanalytical data: *Hl*-DPleB-28 (see **figure S13** for formula). UV/Vis (MeOH, $c = 5.2 * 10^{-5}$): λ_{max} (rel ε) = 237 (1.62), 274 (0.57) nm. ECD ($c = 5.2 * 10^{-5}$); λ_{max} / nm ($\delta\varepsilon$): 208 (-5), 228.1 (24), 283.7 (34), 312.9 (2). ^{1}H-NMR (600 MHz, CD_3OD, 25°): δ[ppm] = 1.76 (*s*, 3H, H_3C-2^1); 1.98 (*s*, 3H, H_3C-13^1); 2.14 (*s*, 3H, H_3C-7^1); 2.39 (*t*, 2H, *J* = 7.32, H_2C-12^2); 2.52 (*m*, 1H, H_AC-3^1); 2.60 (*d*, 1H, *J* = 3.88, H_AC-5); 2.65 (*t*, 1H, *J* = 7.32, H_AC-12^1); 2.75 (*m*, 1H, H_BC-12^1); 2.79 (*t*, 1H, *J* = 6.56, H_BC-3^1); 3.10 (*d*, 1H, *J* = 3.88, H_BC-5); 3.12 (*d*, 2H, *J* = 3.12, H_2C-15); 3.70 (*t*, 2H, *J* = 6.56, H_2C-3^2); 3.77 (*s*, 3H, H_3C-8^5); 4.35 (*t*, 1H, *J* = 3.12, HC-16); 4.42 (*t*, 1H, *J* = 3.88, HC-4); 4.44 (*d*, 1H, *J* = 2.52, H_AC-17^1); 4.64 (*d*, 1H, *J* = 2.52, H_bC-17^1); 4.89 (*m*, 1H, HC-10) superimposed by water; 5.43 (*dd*, 1H, *J* = 2.30 / 11.50, H_AC-18^2); 6.09 (*dd*, 1H, *J* = 2.30 / 17.90, H_BC-18^2); 6.56 (*dd*, 1H, *J* =11.50 / 17.90, HC-18^1); 6.87 and 8.03 are signals corresponding to an unknown impurity; ^{13}C-NMR (600 MHz, CD_3OD, 25°): δ[ppm] = 7.1 (2^1); 7.9 (13^1); 8.2 (7^1); 20.3 (12^1); 28.7 (5); 28.7 (15); 29.6 (3^1); 35.8 (10); 37.3 (12^2); 51.2 (8^5); 55.5 (17^1); 58.8 (16); 59.1 (4); 64.5 (3^2); 66.3 (8^2); 111.6 (7); 114.4 (13); 118.8 (12); 119.4 (18^2); 122.9 (11); 124.3 (8); 125.4 (18^1); 128.7 (18); 129.2 (2); 132.8 (14); 133.1 (6); 154.8 (3); 156.2 (17); 159.8 (9); 170.5 (8^3); 172.8 (19); 175.5 (1); 179.1 (12^3). MS (ESI): m/z (%) = 687.24 (24, $[M+K]^+$); 671.27 (15, $[M+Na]^+$), 651.30 (06), 650.29 (36), 649.29 (100, $[M+H]^+$, $C_{34}H_{41}O_9N_4^+$); MS/MS (18% N_2): m/z (%) = 649.29 (17, $[M+H]^+$, $C_{34}H_{41}O_9N_4^+$); 631.27 (05, $[M+H-H_2O]^+$); 617.27 (100, $[M+H-CH_3OH]^+$); 599.25 (46, $[M+H-H_2O-CH_3OH]^+$); 510.22 (44, [M+H-ring D]$^+$);

Spectroanalytical data: *Hl*-DPxB-31 (see **figure S14** for formula). UV/Vis (MeOH, c = 2.38 * 10^{-5}): λ_{max} (rel ε) = 237 (0.93), 439 (1.06) nm. ECD (c = 2.4 * 10^{-5}); λ_{max} / nm ($\delta\varepsilon$): 285.7 (-23), 309.6 (16), 351.9 (-6). ^{1}H-NMR (700 MHz, CD_3OD, 25°): δ[ppm] = 1.75 (*s*, 3H, H_3C-2^1); 2.13 (*s*, 3H, H_3C-17^1); 2.17 (*s*, 3H, H_3C-13^1); 2.25 (*m*, 2H, H_2C-12^2); 2.47 (*t*, 1H, J = 6.60, H_AC-3^1); 2.59 (2H, H_2C-5); 2.74 (*t*, 1H, J = 6.60, HC-3^1); 2.75 (*m*, 2H, J = 7.44, H_2C-12^1); 3.68 (*t*, 2H, J = 6.60, H_2C-3^2); 3.78 (*d*, 3H, J = 3.00, H_3C-8^5); 4.04 (*dd*, 1H, J = 1.66 / 3.00 HC-8^2); 4.37 (1H, HC-4); 4.63 (*s*, 2H, H_2C-17^1); 4.89 (*m*, 1H, HC-10) superimposed by water; 5.42 (*dd*, 1H, J = 2.30 / 11.50, H_AC-18^2); 6.28 (*dd*, 1H, J = 2.30 / 17.90, H_BC-18^2); 6.48 (*s*, 1H, HC-15); 6.71 (*dd*, 1H, J = 17.50 / 17.90, HC-18^1); 6.87 and 8.03 are signals corresponding to an unknown impurity; ^{13}C-NMR (700 MHz, CD_3OD, 25°): δ[ppm] = 7.2 (2^1); 8.3 (7^1); 8.3 (13^1); 28.6 (5); 29.1 (12^1); 29.5 (3^1); 34.8 (12^2); 35.9 (10); 51.6 (8^5); 52.6 (17^1); 59.1 (4); 61.3 (3^2); 66.1 (8^2); 102.5 (15); 111.9 (7); 118.5 (18^2); 122.4 (12); 123.7 (14); 124.4 (8); 124.7 (18); 125.0 (13); 125.8 (18^1); 128.1 (16); 129.6 (2); 131.6 (11); 133.4 (6); 143.0 (17); 154.7 (3); 170.2 (8^3); 171.5 (19); 175.4 (1); 177.8 (12^3); 190.9 (8^1). MS (ESI): m/z (%) = 685.23 (06, $[M+K]^+$); 669.25 (12, $[M+Na]^+$), 649.28 (06), 648.27 (35), 649.29 (100, $[M+H]^+$, $C_{34}H_{41}O_9N_4^+$); MS/MS (18% N_2): m/z (%) = 647.27 (20, $[M+H]^+$, $C_{34}H_{41}O_9N_4^+$); 629.26 (17, $[M+H-H_2O]^+$); 615.24 (100, $[M+H-CH_3OH]^+$); 597.23 (22, $[M+H-H_2O-CH_3OH]^+$);

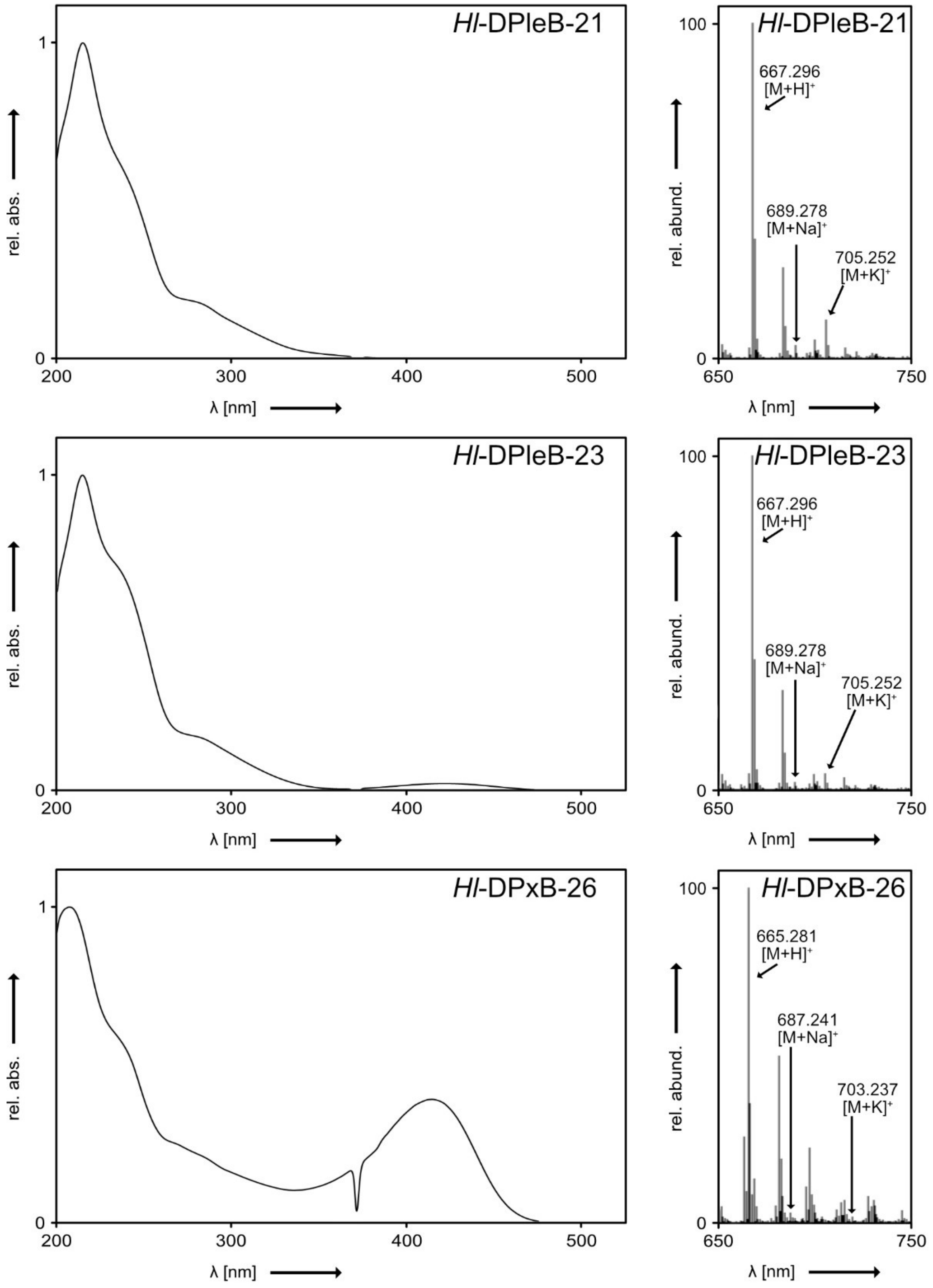

Figure S1a. Online UV/Vis and MS spectra of *Hl*-DPleB-21, *Hl*-DPleB-23, and *Hl*-DPxB-26.

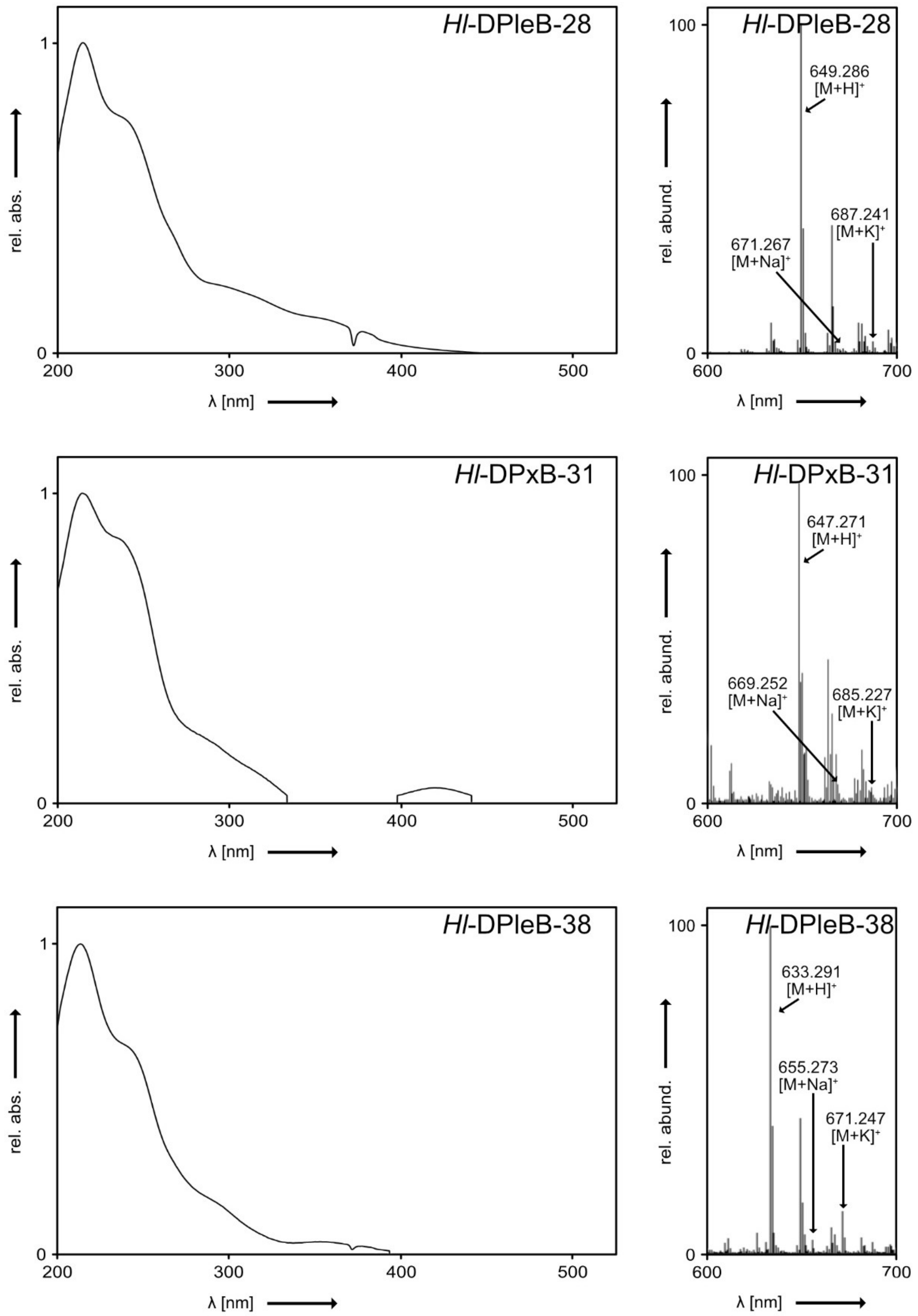

Figure S1b. Online UV/Vis and MS spectra of *Hl*-DPleB-28, *Hl*-DPleB-31, and *Hl*-DPeB-38.

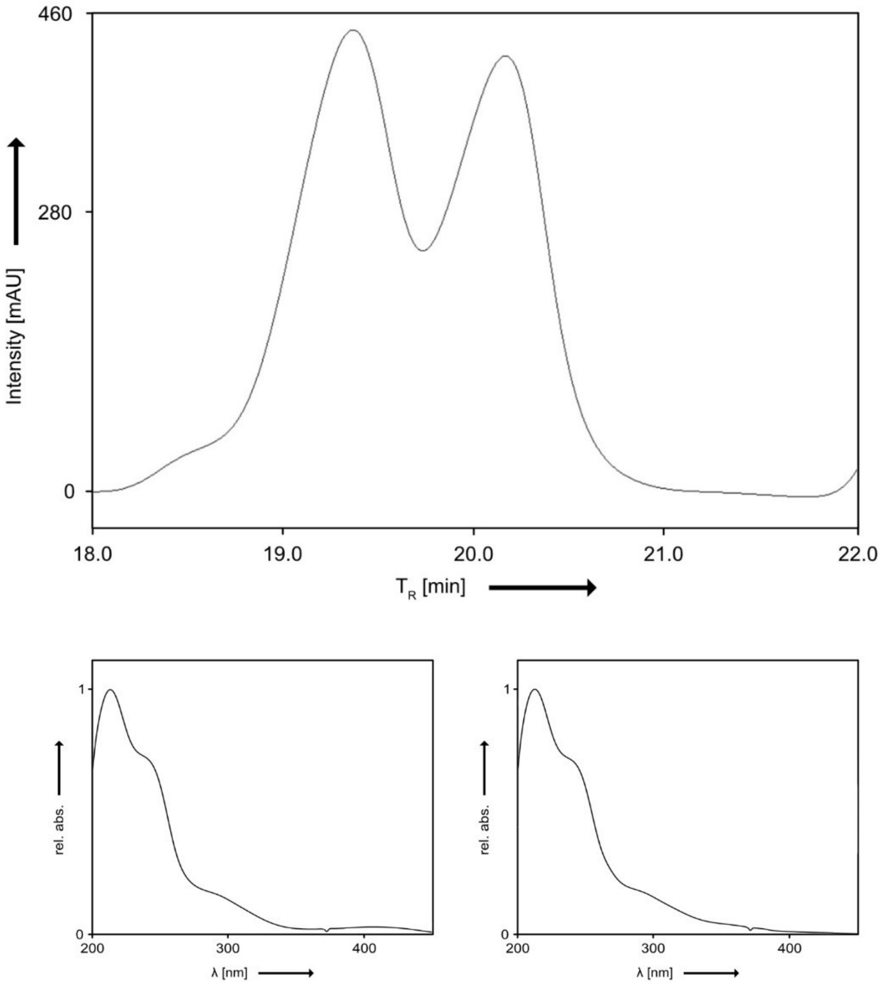

Figure S2. Top: Isocratic HPLC analysis of *Hl*-DPleB-28 fraction, solvent composition A/B, 67/33. The analysis revealed the presence of two isomers. Bottom: Online UV/Vis spectra of the two isomers. For the comparison of NMR data of the two isomers see Table S2.

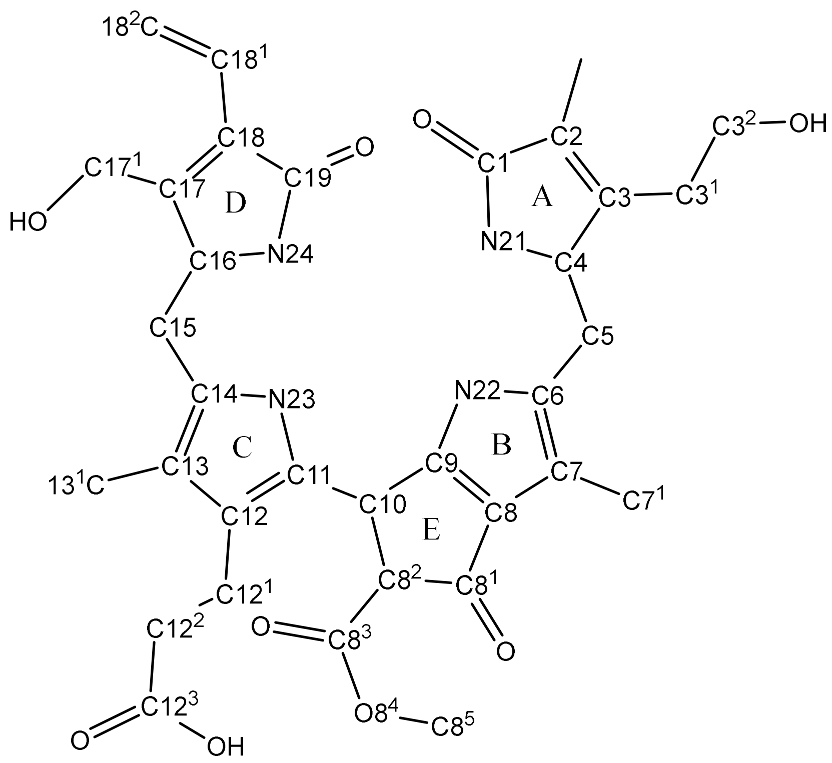

Figure S3. Atom numbering used for the phyllobilin skeleton. [4]

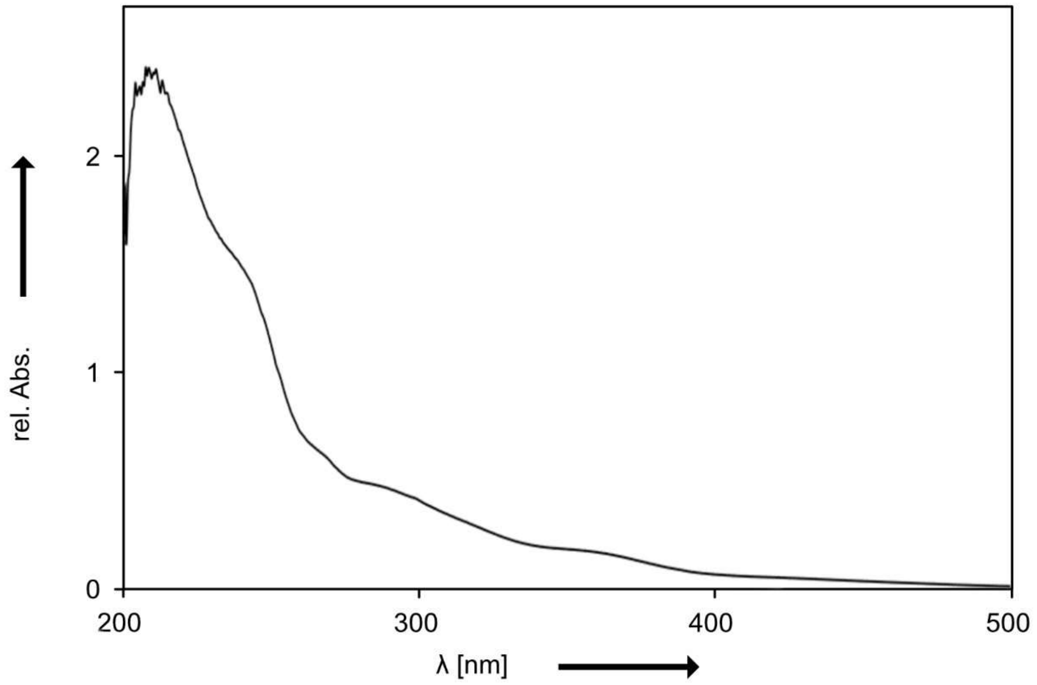

Figure S4. UV/Vis spectrum of *Hl*-DPleB-28 in MeOH.

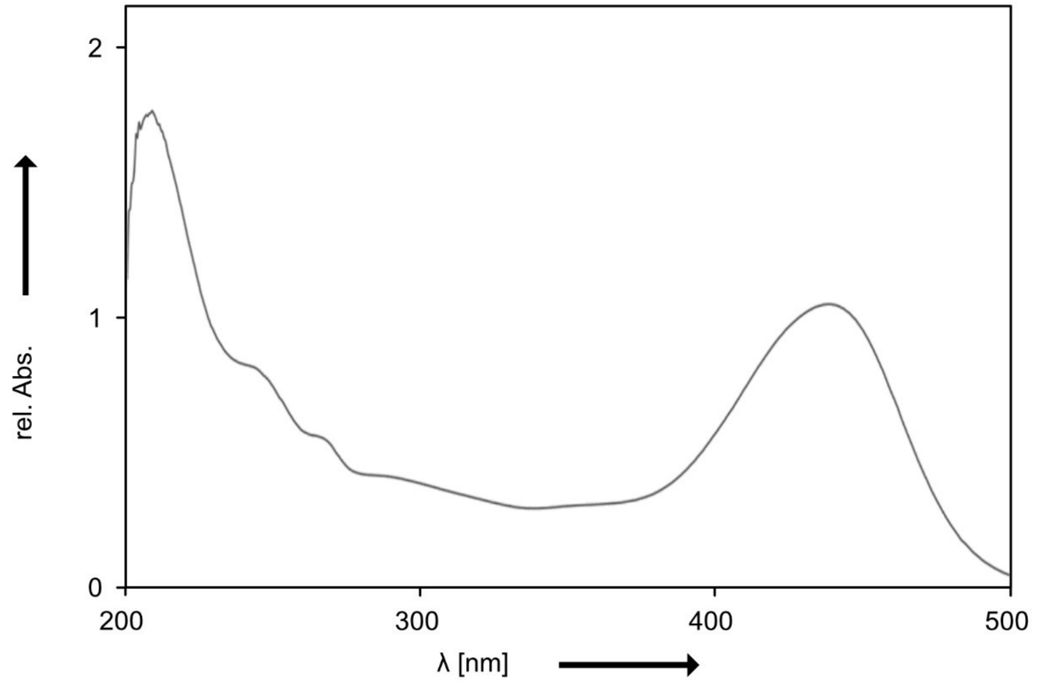

Figure S5. UV/Vis spectrum of *Hl*-DPxB-31 in MeOH.

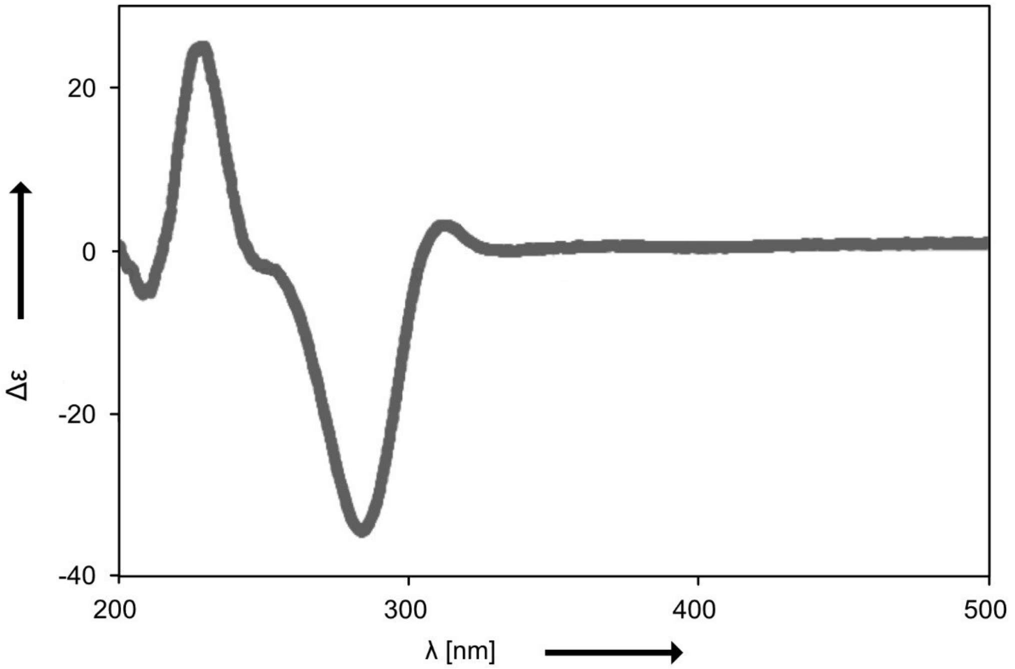

Figure S6. ECD spectrum of *Hl*-DPleB-28 in MeOH.

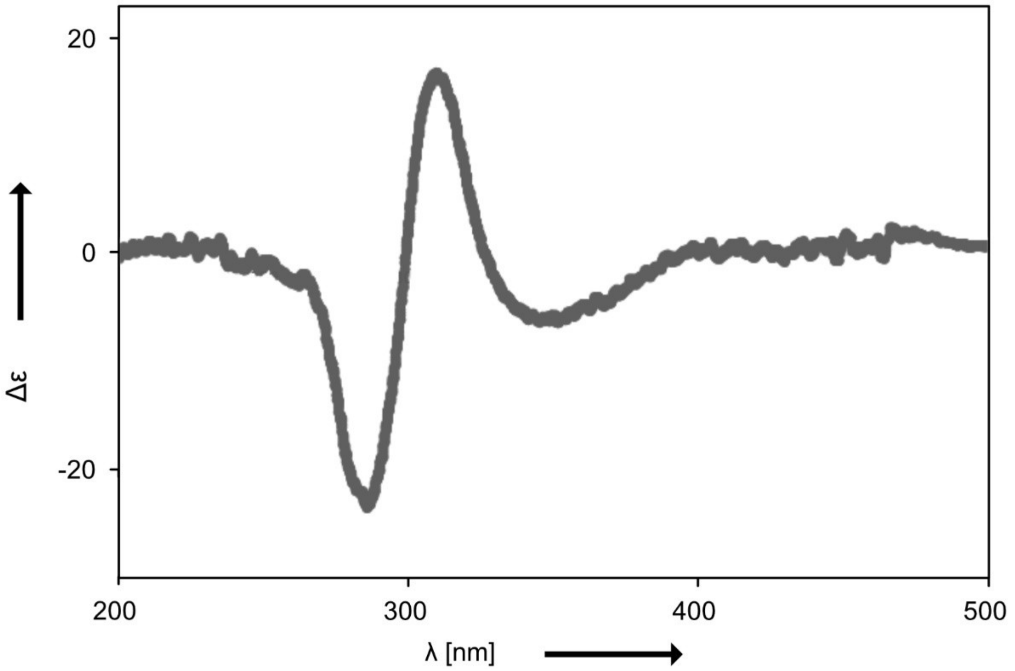

Figure S7. ECD spectrum of *Hl*-DPxB-31 in MeOH.

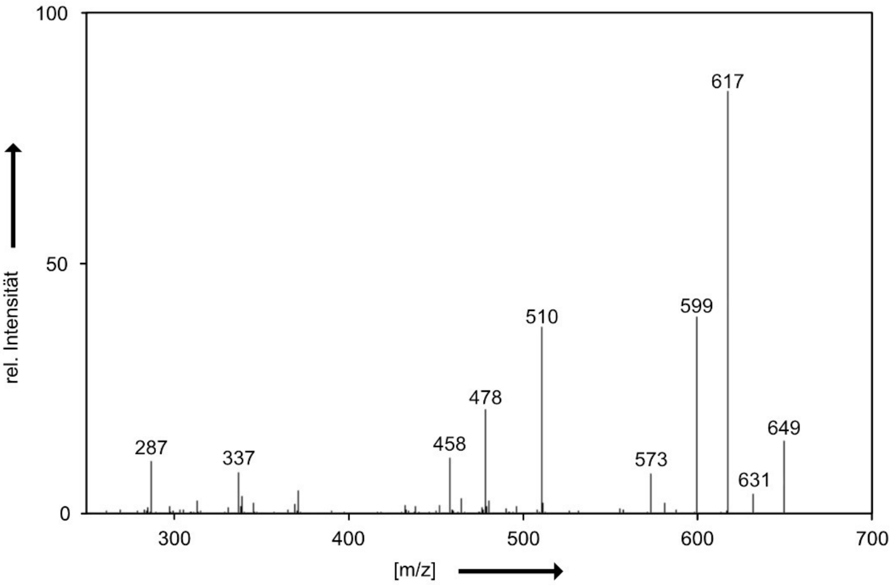

Figure S8. MS^2 analysis of *Hl*-DPleB-28, protonated molecular ion at *m/z* = 649 ($[M+H]^+$); most abundant fragments were observed at *m/z* = 631 ($[M+H-H_2O]^+$), *m/z* = 617 ($[M+H-MeOH]^+$), *m/z* = 599 ($[M+H-H_2O-MeOH]^+$), *m/z* = 573 ($[M+H-MeOH-CO_2]^+$), *m/z* = 510 ($[M+H-Ring D]^+$), *m/z* = 478 ($[M+H-Ring D-MeOH]^+$), *m/z* = 458 ($[M+H-Ring A-H_2O-MeOH]^+$), *m/z* = 371 ($[M+H-Ring D-Ring A]^+$), *m/z* = 337 ($[M+H-Ring D-Ring A-MeOH]^+$), *m/z* = 287 ($[M+H-Ring A-Ring B\&E-H_2O]^+$).

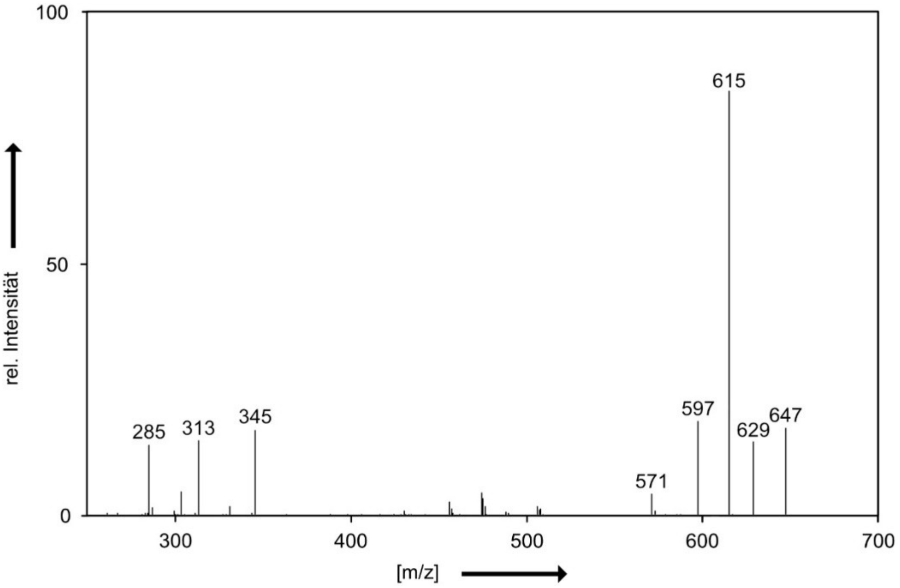

Figure S9. MS^2 analysis of *Hl*-DPxB-31, protonated molecular ion at *m/z* = 647 ($[M+H]^+$); most abundant fragments were observed at *m/z* = 629 ($[M+H-H_2O]^+$), *m/z* = 615 ($[M+H-MeOH]^+$), *m/z*

= 597 ([M+H-H_2O-MeOH]$^+$), *m/z* = 571 ([M+H-MeOH-CO_2]$^+$), *m/z* = 345 ([M+H-Ring D-Ring C)]$^+$), *m/z* = 313 ([M+H-Ring D-Ring C-MeOH]$^+$), *m/z* = 285 ([M+H-Ring A-Ring B&E-H_2O]$^+$).

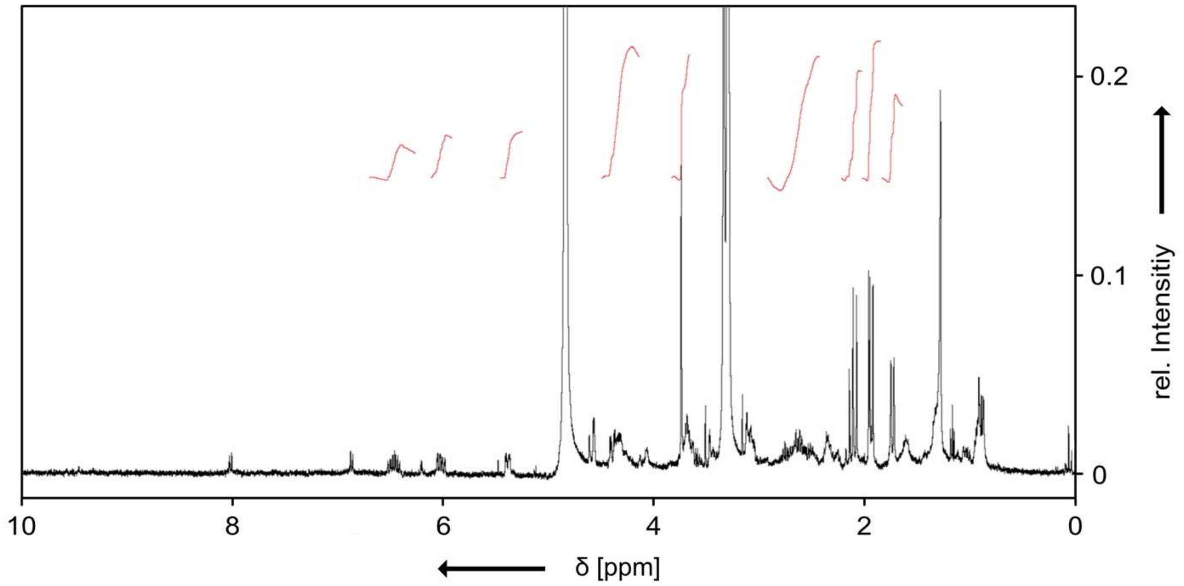

Figure S10. 400 MHz ^{1}H NMR spectrum of *Hl*-DPleB-28 (in CD_3OD, 25°C). Signals at δ_H = 6.87 ppm and δ_H = 8.03 ppm are related to an unknown impurity (see also Figure S11).

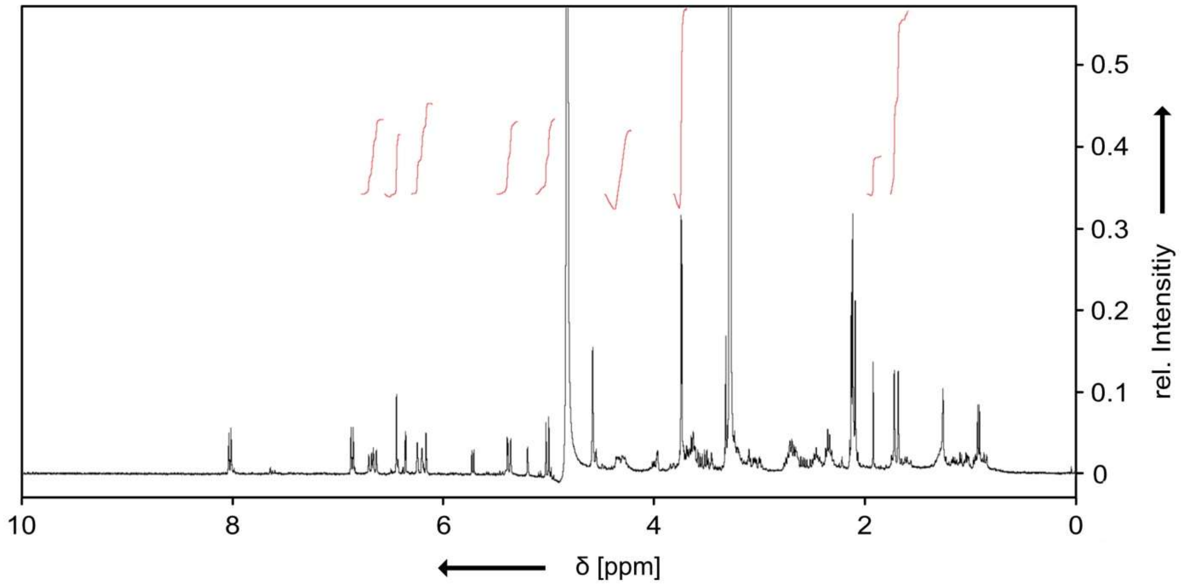

Figure S11. 400 MHz ^{1}H NMR spectrum of *Hl*-DPxB-31 (in CD_3OD, 25°C). Signals at δ_H = 6.87 ppm and δ_H = 8.03 ppm are related to an unknown impurity (see also Figure S10).

Table S1. ^{13}C- and ^{1}H-NMR chemical shift values (CD_3OD, 25°) and signal assignments of *Hl*-DPleB-28 (600 MHz (^{1}H)/150 MHz (^{13}C)) and *Hl*-DPxB-31 (700 MHz (^{1}H)/175 MHz (^{13}C)).

	***Hl*-DPleB-28** **δ^{13}C(ppm)**	***Hl*-DPleB-28** **δ^{1}H(ppm)/J(Hz)**	***Hl*-DPxB-31** **δ^{13}C(ppm)**	***Hl*-DPxB-31** **δ^{1}H(ppm)/J(Hz)**
C 1	175.5		175.4	
C 2	129.2		129.6	
C 2^1	7.1	1.76	7.2	1.75
C 3	154.8		154.7	
C 3^1	29.6	2.79, J = 6.6<br>2.52, J = 6.6	29.5	2.74, J = 6.6<br>2.47, J = 6.6
C 3^2	60.1	3.70	61.3	3.68
C 4	59.1	4.42, J = 3.9	59.1	4.37
C 5	28.7	3.10 / 2.60	28.6	2.59
C 6	133.1		133.4	
C 7	111.6		111.9	
C 7^1	8.2	2.14	8.3	2.13
C 8	124.3		124.4	
C 8^1	-		190.9	
C 8^2	66.3		66.1	4.04, J = 3.0
C 8^3	170.5		170.2	
O 8^4				
C 8^5	51.2	3.77	51.6	3.78
C 9	159.8		-	
C 10	35.8	4.89	35.9	4.89
C 11	122.9		131.6	
C 12	118.8		122.4	
C 12^1	20.3	2.75, J = 7.3<br>2.65, J = 7.3	29.1	2.75, J = 7.4
C 12^2	37.3	2.39	34.8	2.25
C 12^3	179.1		177.8	
C 13	114.4		125.0	
C 13^1	7.9	1.98	8.3	2.17
C 14	123.3		123.7	
C 15	28.7	3.12, J = 3.1	102.5	6.48
C 16	58.8	4.35	128.1	
C 17	156.2		143.0	
C 17^1	55.5	4.64, J = 4.2<br>4.44, J = 1.7	52.6	4.63
C 18	128.7		124.7	
C 18^1	125.4	6.56	125.8	6.71
C 18^2	119.4	6.09, J = 2.3/17.9<br>5.43, J = 2.3/11.5	118.5	6.28, J = 2.0/17.9<br>5.42, J = 2.0/11.3
C 19	172.8		171.5	

Table S2. ^{1}H-NMR chemical shift values of the two isomers of *Hl*-DPleB-28 (CD_3OD, 25°C) extracted from heteronuclear ^{1}H, ^{13}C-HSQC and ^{1}H, ^{13}C-HMBC-spectra (600 MHz (^{1}H)/150 MHz (^{13}C), see also Figure 3).

	δ1H(ppm)		
	Hl -DPleB-28a	*Hl* -DPleB-28b	Δ(ppm)
C 1			
C 2			
C 2^1	1.76	1.74	0.02
C 3			
C 3^1	2.52 / 2.79	2.52 / 2.79	0 / 0
C 3^2	3.70	3.70	0
C 4	4.42	4.42	0
C 5	2.60 / 3.10	3.10	0
C 6			
C 7			
C 7^1	2.14	2.10	0.04
C 8			
C 8^1			
C 8^2	--	--	--
C 8^3			
C 8^4	3.77	3.77	0
C 9			
C 10	4.89	4.74	0.15
C 11			
C 12			
C 12^1	2.65 / 2.75	2.56 / 2.68	0.11 / 0.07
C 12^2	2.39	2.32	0.07
C 12^3			
C 13			
C 13^1	1.98	1.95	0.03
C 14			
C 15	3.12	3.12	0
C 16	4.35	4.33	0.02
C 17			
C 17^1	4.44 / 4.64	4.40 / 4.67	0.04 / -0.03
C 18			
C 18^1	6.56	6.55	0.01
C 18^2	5.43 / 6.09	5.43 / 6.09	0 / 0
C 19			

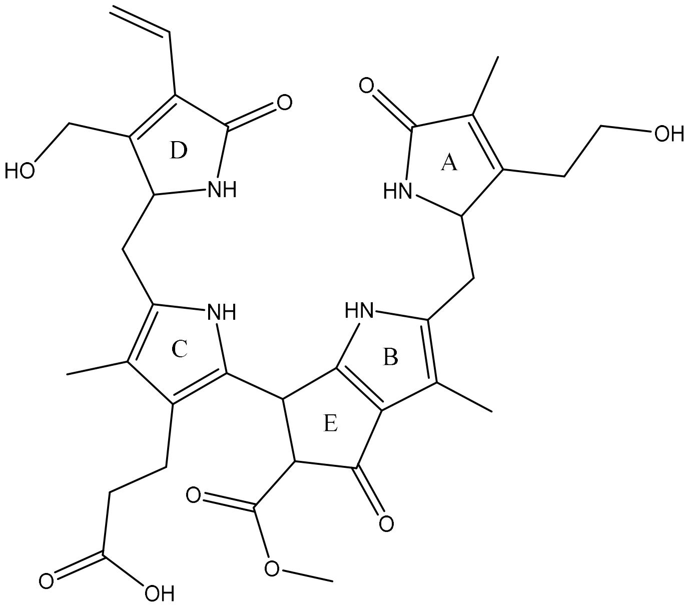

Figure S12. Constitutional formula of *Hl*-DPleB-28.

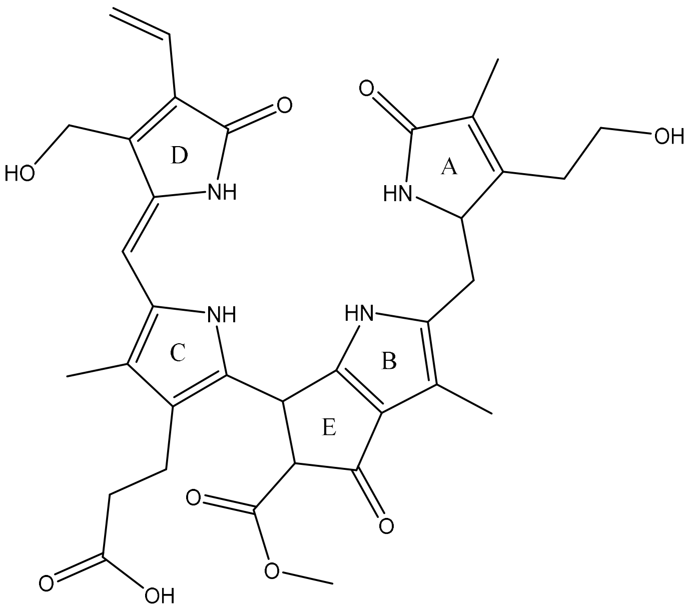

Figure S13. Constitutional formula of *Hl*-DPxB-31.

Testing of antioxidant effects

Reagents and chemicals: Ethanol (EtOH) was purchased from VWR International GmbH (Ismaning, Germany), ultra-pure water (18 MΩ.cm^{-1}) from a Millipore S.A.S Milli-Q Academic system (18,2 MΩ cm^{-1}, Molsheim, France) and hydrogen peroxide (30%) was from Bernd Kraft (Duisburg, Germany). 2,4,6-Tri(2-pyridyl)-s-triazine (TPTZ), iron(III)chloride ($FeCl_3$), quercetin were from Merck (Darmstadt, Germany). Trolox was from Enzo Life Sciences GmbH (Lörrach, Germany), 2´,7´-dichlorodihydrofluoroscein diacetate (H_2DCF-DA) were from Thermo Fisher (Waltham, MA, USA). DMSO was from Carl Roth (Karlsruhe, Germany) and from Sigma-Aldrich, Vienna, Austria, which was also the supplier for quercetin.

DMEM medium was optained from PAN-Biotech (Aidenbach, Germany); Fetal calf serum (FCS) was from PAA Laboratories GmbH (Pasching, Austria).

Spectroscopy: UV/Vis: λ_{max} [nm] (ε_{rel}), Thermo Spectronic Genesys 5 (336001) UV-Visible spectrophotometer. Concentrations of *Hl*-DPxB-31 were calculated using log ε (426 nm) = 4.51[5] and *Hl*-DPleB-28 log ε (237 nm) = 4.49.[6] Tecan SpectraFluor plus microplate reader (F129005).

Nrf-2/ARE Reporter Gene Assay

The CellSensor® ARE-bla HepG2 cell line (Invitrogen - Thermo Fisher Scientific, Vienna, Austria) contains a bacterial β-lactamase gene that is controlled by a ARE response element, which can be induced by the activation of Nrf-2 signalling. β-Lactamase activity is determined by measuring the intensity of fluorescence resonance transfer (FRET) substrate that is generated by enzyme-mediated cleavage. As decribed previously,[7] CellSensor® ARE-bla HepG2 cells were seeded in a 96-well plate (7.5 × 104 cells/ 90 µL/ well) and incubated for 8 h. Cells were either left untreated or treated with the test analytes (Hl-PxB dissolved in water, quercetin dissolved in DMSO) or vehicle control (DMSO). Then, cells were treated with the LiveBLAzer™-FRET B/G substrate (CCF4-AM; Invitrogen- Thermo Fisher Scientific, Vienna, Austria), according to the manufacturer’s protocol. Fluorescence intensity was measured at excitation / emission 414 nm / 460 nm and 414 nm / 538 nm.

Statistical analysis: Results display the mean of at least three independent experiments (mean ± standard deviation), each replicated in at least three repeats, if not declared else. Statistical significance was calculated by one-way analysis of variance with post hoc analysis using Dunnett's multiple comparison test. All statistical analyses were examined with GraphPad Prism 9.

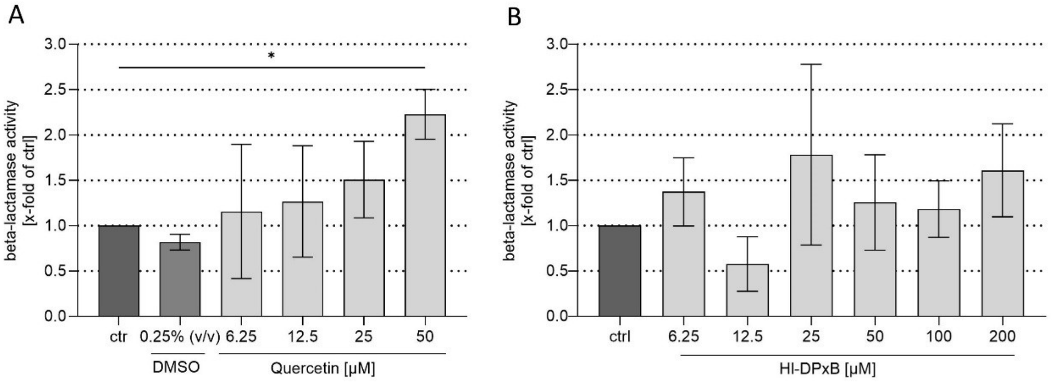

Figure S14. Results of the Nrf-2/ARE reporter gene assay for quercetin (A) and Hl-DPxB (B).

Testing compounds for a potential bitter taste

Reagents and chemicals: Amarogentin was purchased from phytolab (Vestenbergsgreuth, Germany); strychnine, RPMI medium, and DMSO were from Merck (Darmstadt, Germany). The Monarch Total RNA Miniprep Kit, LunaScript RT SuperMix, and Luna Universal Probe qPCR Master Mix were from New England BioLabs (Frankfurt am Main, Germany). Primers were ordered from metabion (Planegg, Germany). Fetal calf serum (FCS) was from ThermoFisher (Waltham, MA, USA).

Multi-taste prediction

	Bitter	Sweet	Umami	Other	in %
Hl-DPxB	60	27	5	8	
Hl-PleB	66	23	1	11	
Cj-PxB	53	44	0	3	
Cj-PleB	57	39	0	4	
Amarogentin	92	6	0	2	
Strychnine	75	9	0	16	
Humulone	89	5	0	5	
Lupulone	94	2	0	4	

Figure S15. Predicted bitterness of phyllobilins isolated from hops, structural representatives of the phyllobilin natural product class (*Cj*-PleB and *Cj*-PxB), known bitter compounds from hops (humulone and lupulone), and the well-known bitter compounds amarogentin and strychnine. Bitterness prediction was performed using VirtualMultitaste, a multi-class prediction tool designed to classify compounds as bitter, sweet, or umami (https://virtuous.isi.gr/#/virtuous-multitaste).[8] The prediction results are presented in a table summarizing the quantitative output (A) and a spider chart illustrating the comparative bitterness profiles of the compounds (B).

mRNA expression levels of human bitter taste receptors

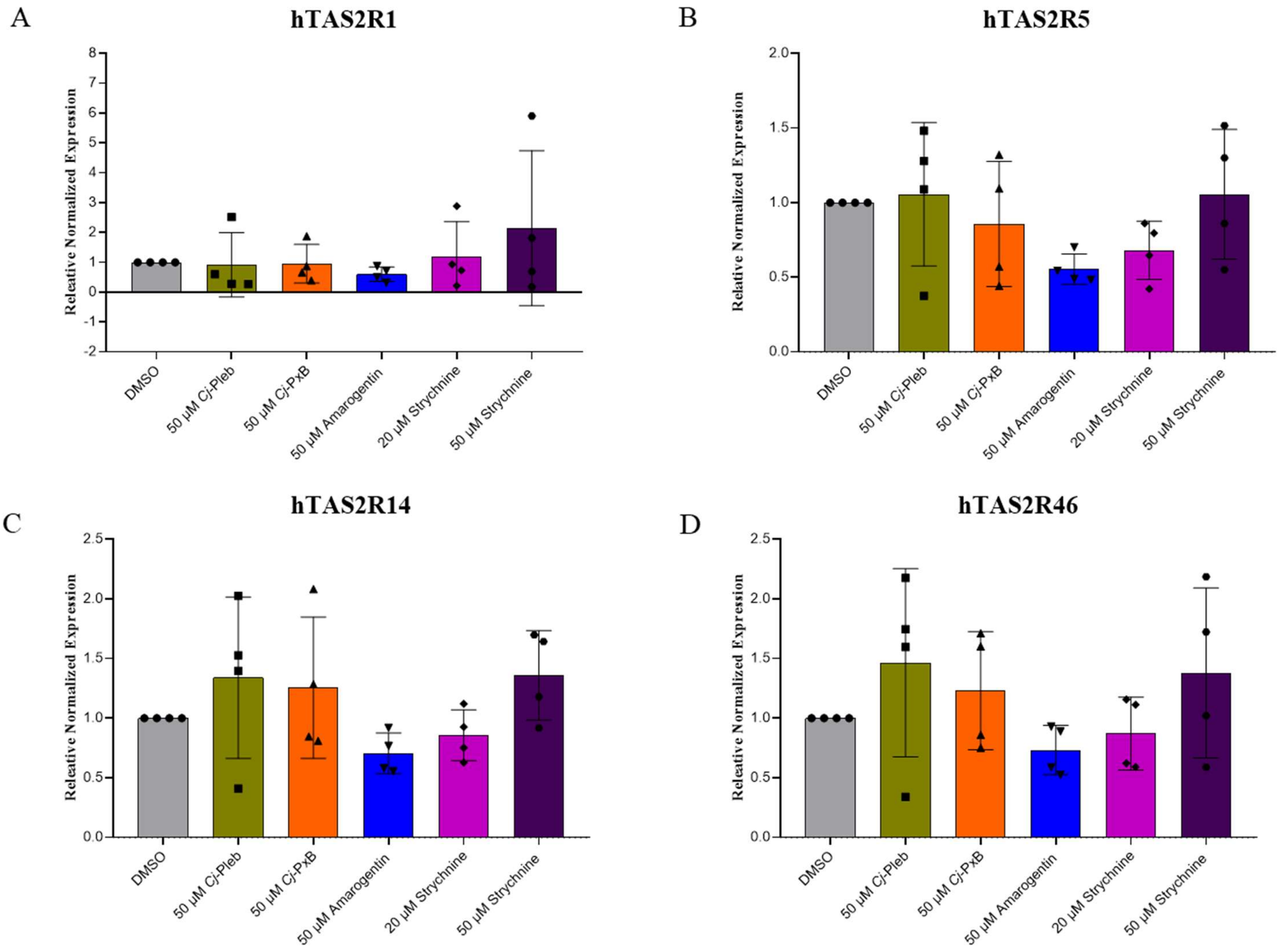

Figure S16. mRNA expression levels of selected human bitter taste receptors hTAS2R1 (A), hTAS2R5 (B), hTAS2R14 (C), and hTAS2R46 (D) measured via qPCR.

For these experiments, structural representatives of phyllobilins, *Cj*-PleB and *Cj*-PxB (isolated from senescent leaves of *Cercidiphyllum japonicum* following the protocol by Karg et al.[9]), as well as the known bitter natural compounds amarogentin and strychnine were used. Briefly, total RNA was extracted from HaCaT cells treated with the compounds or DMSO (control) for 24 hours using the Monarch Total RNA Miniprep Kit. RNA quantity and purity (260/280 nm ratio) were assessed using a NanoDrop spectrophotometer (ThermoFisher, Waltham, MA, USA). Single-stranded cDNA was synthesized from 1 µg of total RNA using the LunaScript RT SuperMix

following the manufacturer's protocol. Primers specific to the human bitter taste receptors were designed with the ExonSurfer tool (https://exonsurfer.i-med.ac.at/).[10] Primer efficiency was determined using serial dilutions of cDNA. qPCR reactions were set up using 50 ng of cDNA, 50 nM of each primer, and the Luna Universal Probe qPCR Master Mix, and performed in duplicate. The qPCR was conducted on a CFX Duet Real-Time PCR System (Bio-Rad, Feldkirchen, Germany) under the following conditions: an initial denaturation step at 95°C for 60 seconds, followed by 50 cycles of 95°C for 15 seconds and 60°C for 30 seconds (fluorescence acquisition), with a melt curve analysis from 60°C to 95°C (1°C increments per step). A final hold at 25°C was applied. mRNA expression levels were calculated as relative normalized expression using the ΔΔCT method, with GAPDH as the reference gene and DMSO-treated cells as the control, which was set to 1. Results represent data from four independent experiments.

References

[1] H.E. Gottlieb, V. Kotlyar, A. Nudelman, NMR chemical shifts of common laboratory solvents as trace impurities, J. Org. Chem. **1997**, 7512-7515.

[2] R. R. Ernst, G. Bodenhausen, A. Wokaun, Principles of Nuclear Magnetic Resonance in One & Two Dimensions, Clarendon Press, **1987**.

[3] K.L. Busch, G. Glish, S.A. McLuckey, Mass spectrometry/mass spectrometry: Techniques and applications of tandem mass spectrometry. VCH publishers, New York, **1988**.

[4] Kräutler, B. *Chem. Soc. Rev.* **2014**, *43*, 6227-6238.

[5] Moser, S, Ulrich, M, Müller, T, Kräutler, B. *Photochemical & Photobiological Sciences* **2008**,7, 1577-1581.

[6] Müller, T, Rafelsberger, M, Vergeiner, C, Kräutler, B. *Angew. Chem. Int. Ed.* **2011**, 50, 10724-10727.

[7] Klein, A.; Wrulich, O.A.; Jenny, M.; Gruber, P.; Becker, K.; Fuchs, D.; Gostner, J.M.; Uberall, F. *BMC Genom*. 2013, 14, 133. https://doi.org/10.1186/1471-2164-14-133.

[8] Androutsos L., Pallante L., Bompotas A., Stojceski F., Grasso G., Piga D., Di Benedetto G., Alexakos C., Kalogeras A., Theofilatos K., Deriu M. A., Mavroudi S., *npj Science of Food* **2024**, 8, 47.

[9] C. A. Karg, P. Wang, F. Kluibenschedl, T. Müller, L. Allmendinger, A. M. Vollmar, et al. European Journal of Organic Chemistry 2020 Vol. 2020 Issue 29 Pages 4499-4509

[10] P. Monfort-Lanzas, E. C. Rusu, L. Parrakova, C. A. Karg, D.-E. Kernbichler, D. Rieder, et al. BMC Genomics 2024 Vol. 25 Issue 1 Pages 594
